# The association between pulse wave velocity and heart failure: a systematic review and meta-analysis

**DOI:** 10.3389/fcvm.2024.1435677

**Published:** 2024-07-23

**Authors:** Zahra Esmaeili, Pegah Bahiraie, Zahra Vaziri, Alireza Azarboo, Amir Hossein Behnoush, Amirmohammad Khalaji, Aida Bazrgar, Pouya Tayebi, Naghmeh Ziaie

**Affiliations:** ^1^ School of Medicine, Tehran University of Medical Sciences, Tehran, Iran; ^2^ School of Medicine, Shahid Beheshti University of Medical Sciences, Tehran, Iran; ^3^ School of Medicine, Babol University of Medical Sciences, Babol, Iran; ^4^ School of Medicine, Shiraz University of Medical Sciences, Shiraz, Iran; ^5^ Department of Vascular and Endovascular Surgery, Rouhani Hospital, Babol University of Medical Sciences, Babol, Iran; ^6^ Clinical Research Development Unit of Rouhani Hospital, Babol University of Medical Sciences, Babol, Iran; ^7^ Department of Cardiology, Babol University of Medical Sciences, Babol, Iran

**Keywords:** arterial stiffness, pulsed wave velocity, heart failure, systematic review, meta-analysis

## Abstract

**Background:**

The arterial stiffness measured by pulsed wave velocity (PWV) is associated with heart failure (HF). However, the effectiveness of arterial stiffness and PWV as prognostic indicators in patients with HFpEF and HFrEF is still unclear. In this systematic review and meta-analysis, we synthesized the prognostic value of PWV and arterial stiffness in HF patients.

**Methods:**

Four databases, including Embase, PubMed, Scopus, and Web of Science, were systematically searched for published studies assessing the relationship between PWV and HF from inception up to August 31, 2023. The Newcastle-Ottawa Scale (NOS) was used to assess the quality of the included studies. The standardized mean difference (SMD) and their corresponding 95% confidence intervals (CI) were used to compare PWV in HF (HFrEF and HFpEF) and controls. Meta-regressions based on age, year of publication, sample size, and gender (male percentage) were also conducted.

**Results:**

The systematic search yielded 5,977 results, of which 58 met our inclusion criteria and 24 were analyzed quantitatively. Studies included 64,687 patients with a mean age of 53.7 years, and 41,803 (67.3%) were male. Meta-analysis of 19 studies showed that PWV was significantly higher in HF patients compared to the controls (SMD 1.04, 95% CI 0.43–1.66, *P* < 0.001, *I*^2^ = 93%). Moreover, nine studies have measured PWV among HFrEF and HFpEF patients and found no significant difference (SMD −0.51, 95% CI −1.03 to 0.02, *P* = 0.057, I2 = 95%). Moreover, increased PWV was linked to an increased chance of developing new-onset HF in individuals with cardiovascular risk factors.

**Conclusions:**

Patients with HF exhibit significantly higher arterial stiffness, as indicated by PWV, compared to the normal population. However, this association was not significant between HFrEF and HFpEF patients. Future research is warranted to establish the potential prognostic role of PWV in HF.

**Systematic Review Registration:**

https://www.crd.york.ac.uk/prospero/display_record.php?ID=CRD42023479683, PROSPERO (CRD42023479683).

## 1 Introduction

Heart failure (HF) is a diverse and potentially fatal syndrome impacting over 60 million people worldwide (1). HF is marked by a high rate of mortality and morbidity, a low quality of life, and a heavy financial and resource strain on healthcare systems (1). The already alarming HF epidemic is anticipated to worsen as the population ages (2). Diabetes, hypertension, and ischemic heart disease are the most prevalent etiologies of HF (3, 4), followed by cardiomyopathies and infections such as viral myocarditis and Chagas' disease (5). Two predominant phenotypes of HF based on left ventricular ejection fraction (EF) have been recognized: HF with preserved EF (HFpEF) and HF with reduced EF (HFrEF). These subtypes differ in their underlying pathophysiology, clinical characteristics, and treatment response (6, 7).

Arterial stiffness, an artery's decreased capacity to expand and contract in response to pressure changes, can predict left ventricular (LV) diastolic dysfunction and is associated with cardiovascular risk (8–11). Greater arterial stiffness is connected to LV diastolic dysfunction and HF with preserved EF (12, 13). The pulse wave velocity (PWV) has been acknowledged as the validated test for examining stiffness in large arteries in the consensus document on ventricular-arterial coupling in cardiac disease (14). PWV is determined by dividing the distance between two points by the time it takes for the pulse to travel between them (2). Increased arterial stiffness, as indicated by elevated PWV, is correlated with poor prognosis in several cardiovascular diseases, particularly in the case of HF (15, 16).

HFpEF has not shown positive responses to conventional pharmacological interventions, except nitrate; hence, there is a growing interest in identifying novel prognostic markers and therapeutic targets for HF (2). This systematic review and meta-analysis aimed to assess the effectiveness of arterial stiffness and PWV as prognostic indicators in patients with HFpEF and HFrEF. By synthesizing data from relevant studies, we seek to elucidate the relationship between arterial stiffness, PWV, and different HF phenotypes, providing insights into their potential utility in risk stratification, management, and therapeutic decision-making for HF patients.

## 2 Materials and methods

This study was written based on the Preferred Reporting Items for Systematic Reviews and Meta-Analyses (PRISMA) 2020 checklist (17). This systematic review and meta-analysis protocol was registered in PROSPERO (CRD42023479683).

### 2.1 Literature search strategy

Four databases, including Embase (Elsevier), PubMed (US National Library of Medicine), Scopus (Elsevier), and Web of Science, were systematically searched for published studies assessing the relationship between PWV and HF from inception up to August 31, 2023. The search terms we used in this study included: “Pulse Wave Analysis” OR “Ankle-Brachial Pulse Wave Velocit*” OR “Pulse Wave Velocity” AND “Heart Failure, Systolic” OR “Heart Failure” OR “Heart Failure, Diastolic” OR “Heart Failure” OR “Cardiac Failure” OR “Congestive Heart Failure” OR “Heart Failure, Reduced Ejection Fraction” OR “Heart Failure, Preserved Ejection Fraction”. Additional details about the medical subject headings, keywords, and entered terms are presented in Supplementary Table S1.

### 2.2 Study selection and eligibility criteria

Two reviewers (Z.E. and Z.V.) assessed each record separately using EndNote 21 software (Tomson Reuters, New York, USA). First, duplicates were removed, and records were screened based on their titles and abstracts. Afterward, the full texts of the studies were assessed; selection of studies adhered to the inclusion and exclusion criteria. The third author (A.A.) acted as the facilitator of agreement meetings to address any potential disputes among reviewers.

Studies were included in this review using the following inclusion criteria: (1) subjects were adults (aged ≥18 years), (2) clinical studies that assessed and compared PWV in HF subtypes, (3) studies that assessed PWV in HF patients and compared them with the normal population, (4) papers that reported baseline PWV in the normal population and assessed for probable incident HF, and (5) studies that reported PWV in different stages of HF. Finally, duplicate publications, studies not reporting PWV, animal studies, case reports, abstracts, and reviews were excluded.

### 2.3 Data extraction and quality assessment

Two reviewers (P.B. and Z.V.) independently extracted the following data from the included studies into a pre-made Excel sheet: first author name, year of publication, study location, study design, sample size, study population (normal or HF), age, gender, and EF. The extracted data were admitted by a third reviewer for probable disparities (A.H.B.).

The Newcastle-Ottawa Scale (NOS) was used to assess the quality of the included studies. The Cochrane Handbook recommends and has created this tool for evaluating the quality of observational studies (18). In cohort studies, there are three key areas to evaluate: selection, comparability, and outcome, with ratings of up to four, two, and three stars; in cross-sectional studies, three aspects were evaluated: selection, comparability, and outcome, with maximus ratings of three, two, and two stars, respectively. A rating of ≥7 is viewed as top quality on this scale. Two separate writers (Z.V. and Z.E.) evaluated the characteristics, and if there was any conflict, a third author (A.K.) settled the matter.

### 2.4 Statistical analysis

Statistical analyses of this study were conducted using the R program [version 4.3.0]. We used Hedges' g standardized mean difference (SMD) and their corresponding 95% confidence intervals (CI) to compare PWV in HF patients and controls and to compare PWV in different HF subtypes, including HFrEF and HFpEF patients (19). We conducted univariable meta-regression based on age, year of publication, sample size, gender (male percentage), and subgroup analysis among HF subtypes based on the locations and the devices used for PWV assessments. The heterogeneity of studies was assessed using Cochrane's Q and Higgins' *I^2^* tests. There was high inter-study heterogenicity if *I^2 ^*> 50% and *P *< 0.1 for the result of the Q test (20). The random-effects model was applied to accommodate the heterogeneity of the enrolled studies (20). *P* < 0.05 reflected statistical significance for all data analyses. Finally, Egger's statistical test and funnel plot were performed for publication bias (21).

## 3 Results

### 3.1 Study selection

**Figure 1:**
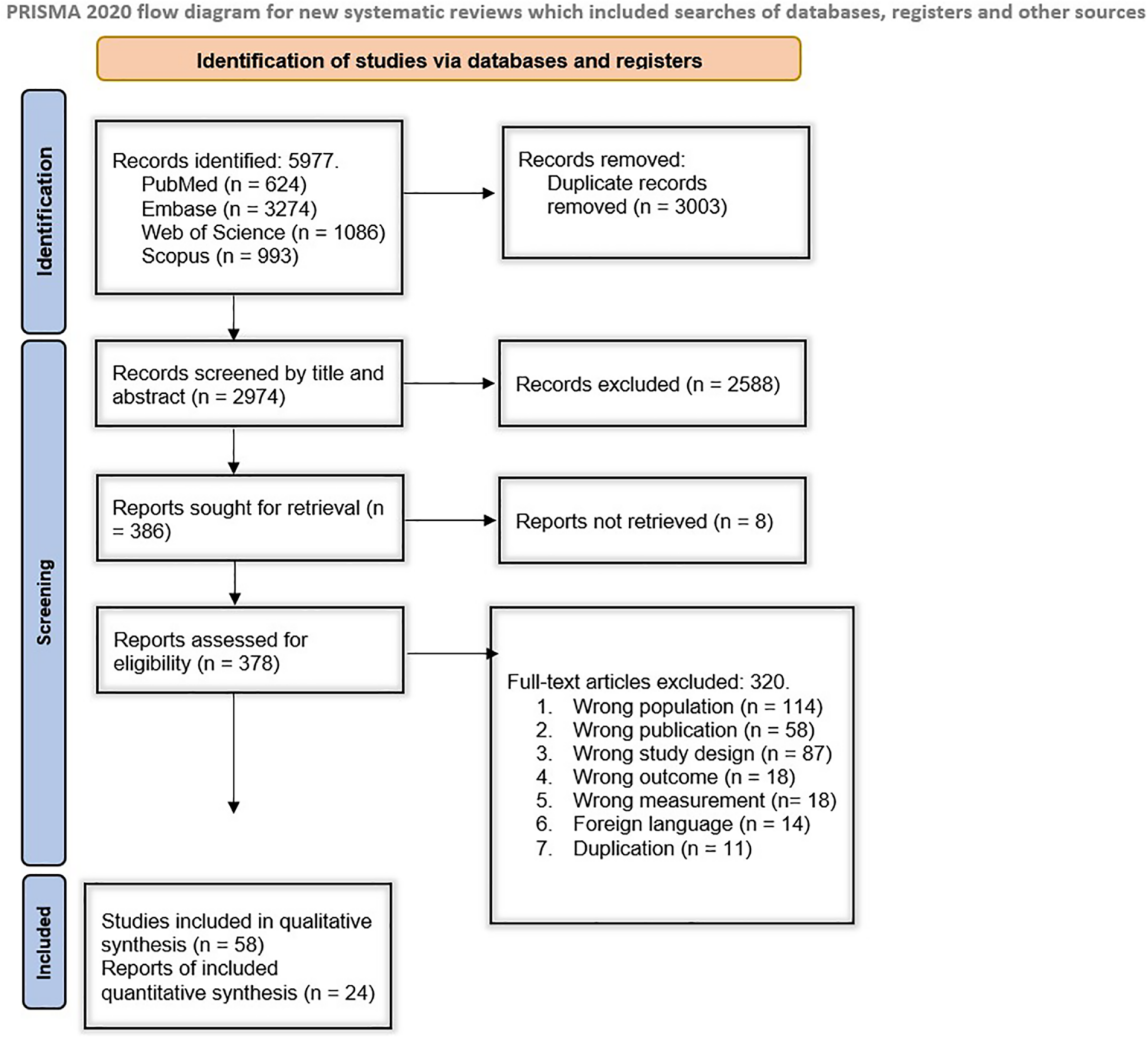
Overview of study selection.

The systematic search of electronic databases yielded 5,977 results, including 624 from PubMed, 3,274 from Embase, 1,086 from Web of Science, and 993 from Scopus. After duplicate removal, 2,974 studies remained. Among those, 2,488 records were excluded during the initial screening based on their title and abstract, and 386 records underwent further full-text screening. The full texts of 378 studies were retrieved and went through an eligibility assessment, from which 319 were excluded. Finally, 58 studies met our inclusion criteria and remained for our qualitative evaluation (22–78). Twenty-four studies were analyzed quantitatively. Figure 1 demonstrates the study selection process in detail.

### 3.2 Baseline characteristics and quality assessment

A summary of the characteristics of the included studies is demonstrated in Table 1. A total of 64,687 patients were included in our study, with sample sizes ranging from 16 to 40,064 participants. Twenty-four studies were conducted in Europe, twenty-four in Asia, nine in America, and one in Africa. The mean age of the population was 53.67 years, and 41,803 (67.27%) were male. The range of mean EF was 21.8 to 68.4%. The carotid-femoral and ankle-brachial arteries were the most frequently assessed anatomical sites for measuring PWV. Carotid-femoral PWV was measured in 25 studies (14, 24, 25, 28, 29, 32–34, 37, 40, 44, 46–49, 51, 53, 54, 57, 61, 65–67, 70, 72, 77, 78), while 13 studies reported ankle-brachial PWV measurements (38–40, 43, 55, 56, 58, 64, 71–75). Of the included studies, 47 had a high-quality score with an overall score of seven or higher based on the NOS tool, while six and five studies scored 6 and 5, respectively (Supplementary Table S2).

**Table 1:** Baseline characteristics of included studies assessing PWV in HF.

Study	Year	Location	Population	Sample size	Age	Male (%)	Ejection fracture	Main findings
Dohaei et al. (22)	2017	Iran	Patients with advanced HF (EF < 30)	50	45 ± 16	76	21.8 ± 8.9	In patients with advanced systolic HF, PWV may not be a good prognostic factor and does not have any added value over previous well known prognostic factors.
El Fol et al. (23)	2022	Egypt	Acute decompensated HFrEF	100	51.6 ± 6.1	80	NA	Central arterial stiffness indices in patients with HFrEF were significantly lower in the compensated state than in the decompensated state.
Shah et al. (24)	2009	UK	HF of underlying IHD or of DCM	39	52.85 ± 15.62	71.7	NA	arterial stiffness as assessed by carotid femoral PWV is increased in heart failure with IHD, but not DCM.
Balmain et al. (25)	2007	UK	CHD	36	73.6 ± 7.4	47.2	51.3 ± 18.2	marked increase in vascular stiffness in HFpEF than in HFrEF.
Spronck et al. (26)	2021	USA	Subjects with and without heart failure	154	64.92 ± 10.9	94.8	NA	The findings support PWV as the primary arterial stiffness metric for outcome prediction.
Bonapace et al. (27)	2013	Italy	Outpatients with diagnosed HF	156	65.5 ± 13.2	82.7	33.4 ± 9	Increased aortic PWV independently predicted adverse clinical outcomes among patients with HF.
Buleu et al. (28)	2019	Romania	IHD with reduced LVEF	120	67.46 ± 8.97	49.16	41.05 ± 14.35	PWV as indicator of arterial stiffness was significantly modified in ischemic HF compared to control.
Chasikidis et al. (29)	2022	Greec	Permanent inhabitants of Corinth	202	71.5 ± 10.98	64.35	47.5 (35–60)	signifying PWV and arterial stiffness as possible diseases and clinical status modifiers in HF patients.
Liu et al. (30)	2013	China	Normal subjects and HF patients	100	60.8 ± 8.49	52	48.8 ± 14.5	For the heart failure patients, PWV was significantly higher and peripheral arterial volume distensibility was significantly lower.
Coksevim et al. (31)	2020	Turkey	Patients with HFrEF	46	70.6 ± 7.9	76.1	28.1 ± 5.0	PWV were increased after cardiac resynchronization therapy.
Ali et al. (32)	2023	UK	Healthy controls, HTN, HTN + DM, HFpEF, HFrEF	94	76.79 ± 5.42	51	59.45 ± 13.78	Support the concept of HFpEF as a disease of the vasculature with increased arterial stiffness that is driven by vascular ageing and comorbidities.
Desai et al. (33)	2009	USA	HFpEF with HTN and healthy normotensive controls	53	65.13 ± 12.18	47.1	68.37 ± 9.07	Patients with heart failure and preserved ejection fraction have increased central aortic stiffness.
Anastasio et al. (34)	2022	Italy	HF patients hospitalized for acute decompensation	199	73.6 ± 16.6	61.3	40.97 ± 12.99	aPWV proved to be an independent factor associated with free-event survival.
Fehérvári et al. (36)	2021	Romania	Hospitalized patients with HFrEF	78	65.8 ± 1.41	73.1	31.85 ± 9.40	PWV high group was older and had higher intima-media thickness, higher incidence of hypertension and higher left ventricular ejection fraction
Fehérvári et al. (35)	2021	Romania	Hemodynamically stable systolic heart failure (EF < 40%)	40	63 ± 12.9	80	NA	increased stiffness was found to be correlated with the general risk factors of arterial involvement
Feola et al. (14)	2019	Italy	Patients with acute heart failure	101	68 ± 13.9	62	NA	PWV proved to be different in HF patients in comparison with CVRF/healthy population
Fantin et al. (37)	2022	Italy	Hospitalized patients affected by HF	41	85.68 ± 5.5	34.1	NA	Cf -PWV is a valid predictor of 30-day readmission
Kim et al. (38)	2023	Republic of Korea	HF and healthy controls	235	67 ± 12.9	60	52.98 ± 18.44	Although arterial stiffness was increased, its association with LV diastolic function was attenuated in HF patients compared to control subjects
Hsu et al. (39)	2010	Taiwan	Patients arranged for echocardiographic examination	267	57.49 ± 13.5	55.8	56.04 ± 17.89	There was no positive correlation between echocardiographic LV diastolic parameters and ba-PWV.
Huang et al. (40)	2019	Taiwan	Acute HF	2,907	75.78 ± 13.24	67.62	53.17 ± 19.07	subjects with HFmrEF were characterized with increased pulsatile hemodynamics, including PP, arterial stiffness and wave reflection.
Ibrahim et al. (41)	2011	USA	Heart failure with normal ejection fraction	16	71.5 ± 6.62	12.2	58.5 ± 9.1	Heart failure with normal EF is associated with impaired LV diastolic function and significant ventricular and aortic stiffening
Arnold et al. (42)	1991	Canada	CHF	67	54.11 ± 9.9	NA	NA	Alterations in brachial artery function were present in patients with moderate and severe CHF.
Zhang et al. (43)	2016	China	Hypertension and diastolic heart failure	116	65.15 ± 3.09	41.3	61.94 ± 5.61	Arterial stiffness is an independent risk factor for early mild DHF in elderly patients with hypertension.
Kim et al. (44)	2013	Republic of Korea	HF: transition from ADHF to CCHF	55	65.4 ± 12.6	46	39.30 ± 8.03	Central and upper-extremity PWVs improved as patients transitioned from ADHF to CCHF
Alem and Alshehr (45)	2020	Saudi Arabia	Compensated CHF with reduced or preserved EF	73	55.9 ± 11.6	79.5	43.0 (32.5, 55.0)	A-PWV was not found to be a significant predictor for LVMI.
Mitchell et al. (46)	2001	Canada	CHF with preserved or impaired LVEF	68	58.64 ± 10.12	80	NA	CF-PWV did not differ whereas CR-PWV was lower in CHF group.
Steinberg et al. (47)	2023	Georgia	HFrEF and healthy controls	261	53.55 ± 11.34	52	NA	Compared with controls, participants with heart failure with reduced ejection fraction exhibited similar carotid-femoral pulse wave velocity.
Pietschner et al. (48)	2022	Germany	Patients with and without CHF	223	60.12 ± 13.01	68	NA	In CHF patients vascular remodeling and functional impairment were observed compared with controls.
Salvi et al. (49)	2018	Italy/Australia/China	Healthy volunteers and heart failure patients	104	54.83 ± 18.37	62.5	51.9 ± 15.5	Arterial tonometry could be considered a useful method for the evaluation of left ventricular performance and improve the calculation of pulse wave analysis.
Demir et al. (50)	2013	Turkey	NYHA stage III–IV patients	98	59 ± 11	77.5	27.35 ± 3.03	During decompensation, AIx and PWV were greater than when the same patients were adequately treated.
Giannitsi et al. (51)	2020	Greece	Hospitalized patients due to AHFS	100	70 ± 11	78	35 (27, 45)	Increased aortic stiffness may predict mortality and HF re-hospitalizations in AHF patients.
Parragh et al. (52)	2019	Austria	Patients with suspected coronary artery disease	183	59.76 ± 11.05	91.8	57.56 ± 23.46	Decreased measures of pulsatile function may be caused by impaired systolic function and altered interplay of left ventricle and vascular system.
Sung et al. (53)	2011	Taiwan	AHFS	80	73.21	82.5	46.15 ± 17.1	Suboptimal recovery of the perturbations of the pulsatile hemodynamics in AHFS may relate to adverse short-term outcomes.
Sung et al. (54)	2012	Taiwan	Hospitalized AHFS	120	71.94 ± 14.3	83.3	42.13 ± 15.1	CF-PWV, significantly independently predicted events in patients hospitalized due to AHFS.
Takae et al. (55)	2019	Japan	HFrEF	185	67.5 ± 11.74	76.2	40.4 ± 9.2	Identifying complications of PAD and measuring ba- PWV values in HFrEF patients were useful for predicting their prognosis.
Tokitsu et al. (56)	2017	Japan	Hospitalized patients with heart failure	502	71.7 ± 9.4	56.2	62.7 ± 5.8	Prognostic significance of ba-PWV values and the utility of ABI devices in risk stratification of HFrEF patients.
Tartière et al. (57)	2005	France	CHF patients	135	65.6 ± 13.3	75.5	35.9 ± 19.1	In subjects with heart failure and low EF, CF-PWV is strongly influenced by simple hemodynamic parameters.
Meguro et al. (58)	2009	Japan	HF patients	72	68 ± 14	56.9	53 ± 18	Elevated arterial stiffness is a risk factor for re-admission or cardiac death of HF patients.
Regnault et al. (59)	2013	France	Patients with HF in the setting of acute MI	306	61 ± 11	74	34.4 ± 5.2	Increased aortic stiffness, assessed by PWV, contributes significantly to cardiovascular death.
Ryabov et al. (60)	2012	Russia	CHF	55	65.8 ± 9.6	53	53.1 ± 6.2	Stiffness of the main arteries is increased in patients with CHF and preserved LV EF after STEMI.
Hashmath et al. (61)	2018	USA	Adults referred for a cardiac MRI study	348	61.7	89.3	54.53	DP-uc-MGP levels are independently associated with large artery stiffening in HF and that warfarin use is associated with arterial stiffness.
Bonapace et al. (62)	2016	Italy	Stable CHF in sinus rhythm	77	62.38 ± 9.6	79	34.1 ± 7.9	Increased aortic stiffness and LV diastolic dysfunction strongly predict the development of incident AF in patients with systolic CHF.
Kaiser et al. (63)	2001	USA	Patients with NYHA class II to IV heart failure	36	51.5 ± 2	75	NA	Brachial arterial wall-to-lumen ratio is increased with a trend toward reduced arterial stiffness in HF.
Sun et al. (64)	2023	China	HFpEF	94	71.74 ± 11.4	45.7	61 ± 4.9	The association of visceral fat with ba-PWV in HFpEF group may be partly accounted for SBP or PP.
Pugliese et al. (65)	2022	Italy	Patients referred for dyspnea or cardiovascular checkup	466	61 ± 4.9	62.2	60.2 ± 16.5	Cf-PWV and aa-PWV were significantly higher in HFpEF than in HFrEF and controls.
Radaelli et al. (66)	2010	Italy	CHF/CAD/healthy controls	89	62.6 ± 1.4	NA	49.9 ± 11.1	PWV was higher in CAD patients than control. CHF patients differed from both controls and CAD patients for lower ejection fraction, lower DBP and higher PWV.
Radaelli et al. (67)	2014	Italy	CHF/CAD/healthy controls	32	61.8 ± 7.6	NA	51.3 ± 11.7	Hemodynamic characteristics of CAD and CHF patients were like those of CNT, except for PWV and baroreflex sensitivity that were similarly increased and reduced, respectively.
Satoshi Suzuki (68)	2018	Japan	Hospitalized HF	221	64.4 ± 13.1	71	45.6 ± 17.01	Severe SDB is associated with elevated arterial stiffness and may be related to the pathophysiology of HF, especially in HFpEF patient.
Tokitsu et al. (69)	2016	Japan	HFpEF	512	71.7 ± 9.4	56.3	62.7 ± 5.8	PP values in HFpEF patients had a strong and significant positive correlation with PWV.
Trembach et al. (70)	2018	Russia	Patients with CHF	87	63 ± 7	NA	34 ± 6	A significant negative correlation between BRS and age, NT-pro-BNP level, and PWV.
Aisu et al. (71)	2016	Japan	Adults with HF risk factors	456	71 (61–76)	68	64 (56–69)	Deterioration of ba-PWV was associated with hospitalization for new-onset HF.
Lee et al. (72)	2021	South Korea	Patients with high cardiovascular risk	3,034	59.2 ± 11.6	54.6	NA	Among arterial stiffness indices, brachial PP, cf-PWV, and central PP were better predictors of HF than ba-PWV.
Cong et al. (73)	2015	China	Patients with acute dyspnea	111	64.2 ± 11.5	47.7	67.3 ± 6.1	Adding the ba-PWV to the diagnostic indicators of the 2007 ESC consensus statement could increase the accuracy of predicting HFpEF.
Zheng et al. (74)	2023	China	Individuals who took part in at least one health evaluation	40,064	48.81 ± 12.67	73.4	NA	Arterial stiffness was positively associated with a higher risk of new-onset HF.
Kang et al. (75)	2010	China	General middle and aged population	1,764	58 ± 12.3	31.6	64.9 + 3.3	The increased arterial stiffness is associated with early mild DHF in a general middle and aged population.
Heffernan et al. (76)	2022	USA	Middle-aged men and women	6,814	NA	47.1	NA	Those in the highest quartile of e-PWV had a significantly higher risk of HF, HFrEF, and HFpEF.
Tsao et al. (77)	2016	USA	Participants without clinical HF	2,267	61.4 ± 9.1	NA	NA	Greater aortic stiffness was associated with increased risk of HF.
Weber et al. (78)	2013	Austria	Patients with dyspnea with LVEF > 50%	369	63.42 ± 9.65	68.8	67.8 ± 9.23	aortic stiffness had diagnostic capacities for HFPEF.

Numbers are presented as mean ± standard deviation, median (interquartile range), or percentage.

HF, heart failure; EF, ejection fraction; HFrEF, heart failure with reduced ejection fraction; HFpEF, heart failure with preserved ejection fraction; PWV, pulse wave velocity; a-PWV, aortic pulse wave velocity; cf-PWV, carotid-femoral pulse wave velocity; ba-PWV, brachial-ankle pulse wave velocity; cr-PWV, carotid-radial pulse wave velocity; NA, not applicable; IHD, ischemic heart disease; DCM, dilated cardiomyopathy; CHD, chronic heart disease; LV, left ventricle; DM, diabetes mellites; HTN, hypertension; CVRF, cardio vascular risk factors; DHF, decompensated heart failure; ADHF, acute decompensated heart failure; CCHF, chronic compensated heart failure; LVMI, left ventricular mass index; NYHA, New York Heart Association; AIx, augmentation index; AHFS, acute heart failure syndromes; MI, myocardial infraction; STEMI, ST-segment elevation myocardial infraction; MRI, magnetic resonance imaging; AF, atrial fibrillation; CAD, chronic artery disease; DBP, diastolic blood pressure; SDB, mean systolic blood pressure; PP, pulse pressure; NT-pro-BNP, N-terminal pro–B-type natriuretic peptide; Dp-ucMGP, Dephosphorylated, uncarboxylated matrix Gla protein; ESC, European Society of Cardiology.

### 3.3 PWV in HF vs. Normal Population

**Figure 2:**
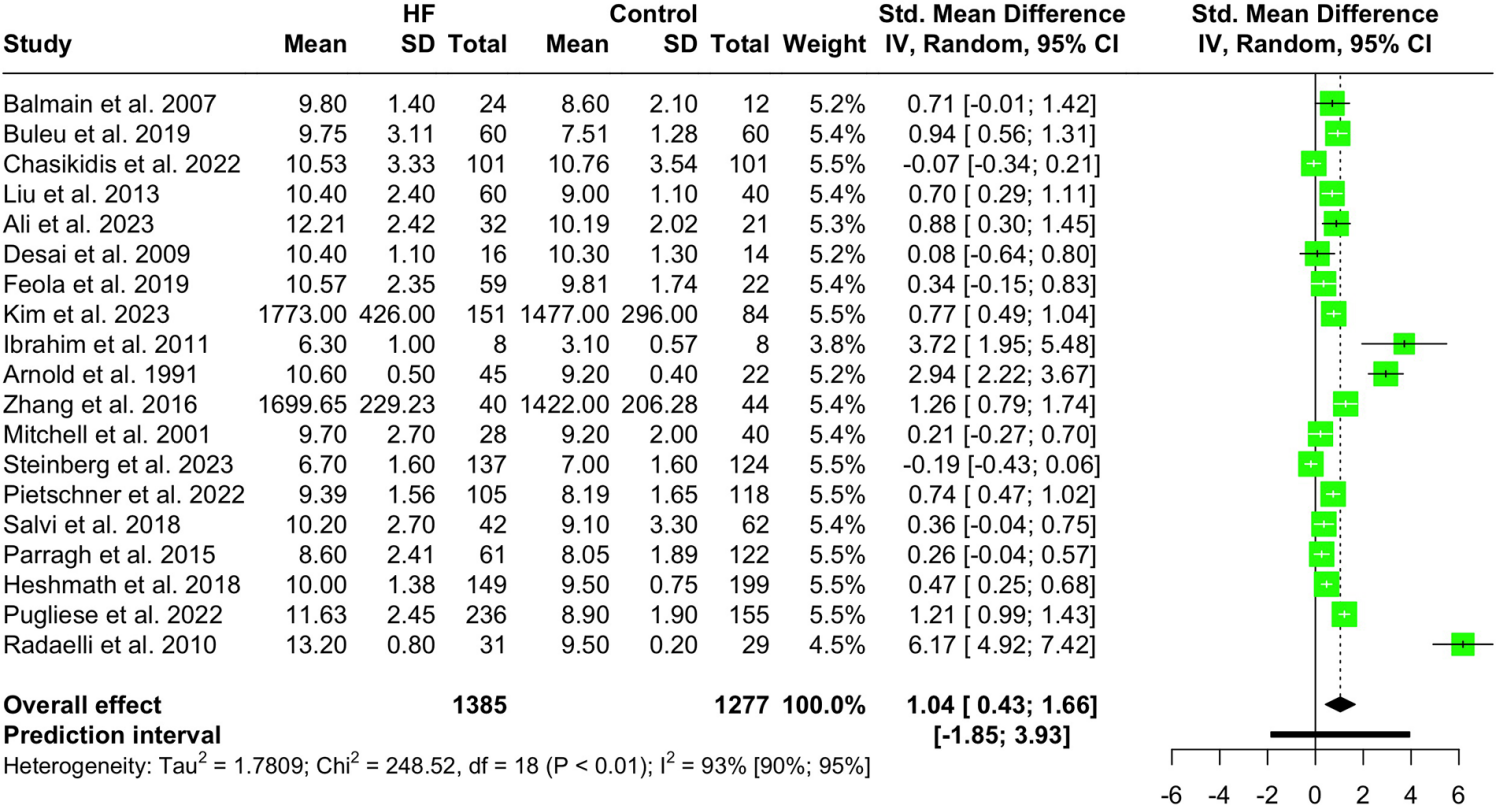
Forest plot showing the PWV difference in HF vs. normal population.

**Figure 3:**
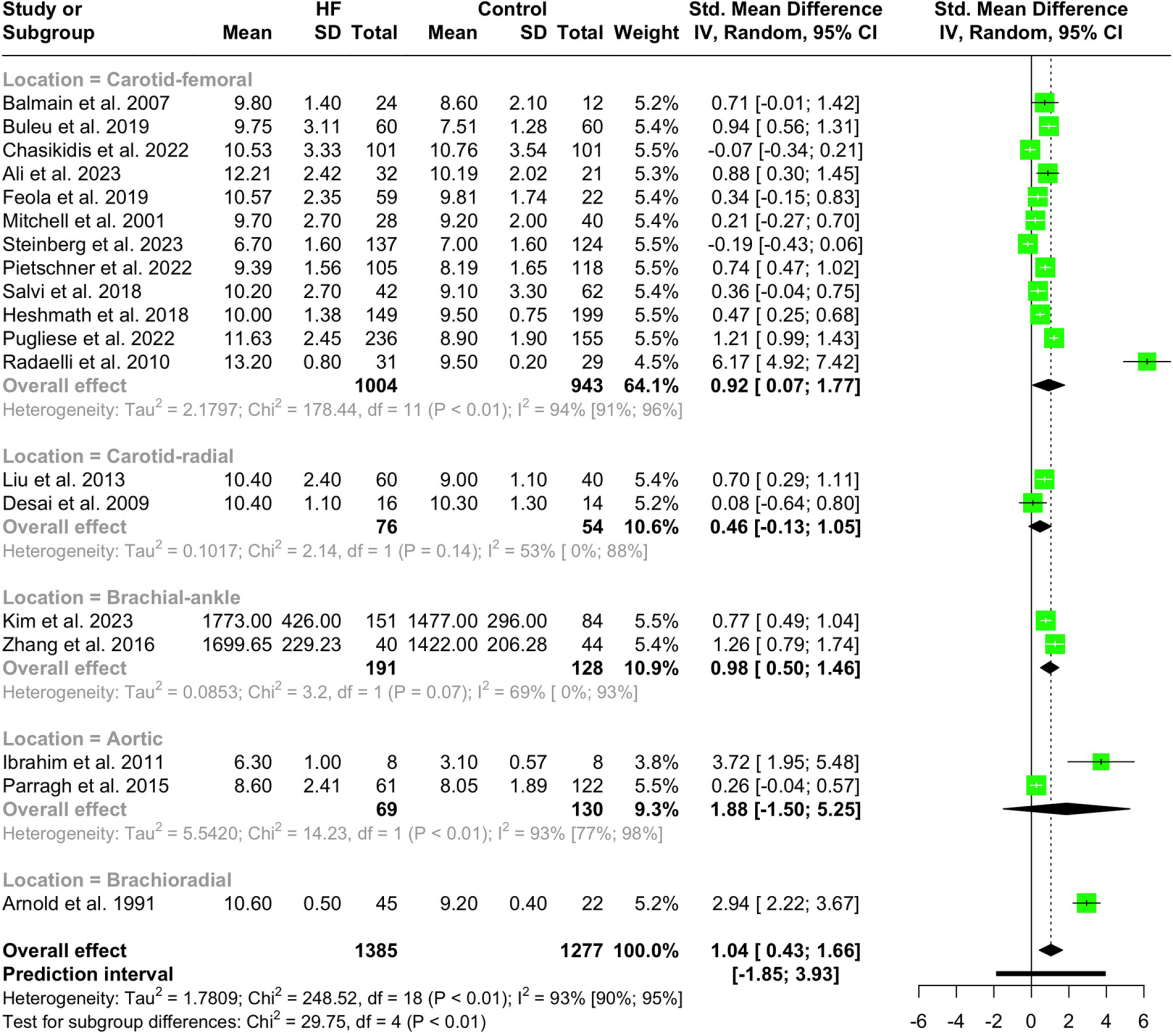
Subgroup analysis of PWV in HF vs. normal population based on the location of PWV measurement.

Nineteen studies with a total population of 2,662 patients measured PWV in HF and healthy individuals and were included in the meta-analysis (14, 25, 28–30, 32, 33, 38, 41–43, 46–49, 52, 61, 65, 66). The results showed that PWV was significantly higher in HF patients compared to the controls (SMD 1.04, 95% CI 0.43 to 1.66, *P* < 0.001), as depicted in the forest plot in Figure 2. Subgroup analysis was conducted based on the location and the devices used for the PWV measurement. As demonstrated in Figure 3, the pooled effect estimates were significant across the carotid-femoral, ankle-brachial, and brachioradial subgroups (SMD 0.92, 95% CI 0.07–1.77; SMD 0.98, 95% CI 0.50–1.46; SMD 2.94, 95% CI 2.22–3.67, respectively). The findings of the analysis based on the devices remained significantly different among studies using SphygmoCor and non-SphygmoCor devices (SMD 0.46, 95% CI 0.17–0.74; SMD 1.79, 95% CI 0.30–3.28) (Supplementary Figure S1).

Addressing high heterogeneity in our initial analysis (*I*^2^ 93%, 95% CI 90.1%–94.7%, *P* < 0.01), five outlier studies were omitted (29, 41, 42, 47, 66). The remaining reports, including 1,385 HF patients and 1,277 controls, showed that PWV was significantly higher among HF patients compared to controls (SMD 0.66, 95% CI 0.47–0.85, *P* < 0.0001, *I*^2^ 75%) (Supplementary Figure S2). The findings of the sensitivity analysis are demonstrated in Supplementary Figure S3, indicating that the pooled SMD estimate was not significantly modified.

Meta-regression revealed a significant association between gender and the PWV in two groups (*β* −0.0166, 95% CI −0.0315 to −0.0017], *P* = 0.02). However, no significant associations were found between the effect size and other investigated variables, including age, year, and sample size (Supplementary Table S3 and Figures S4–S7).

**Figure 4:**
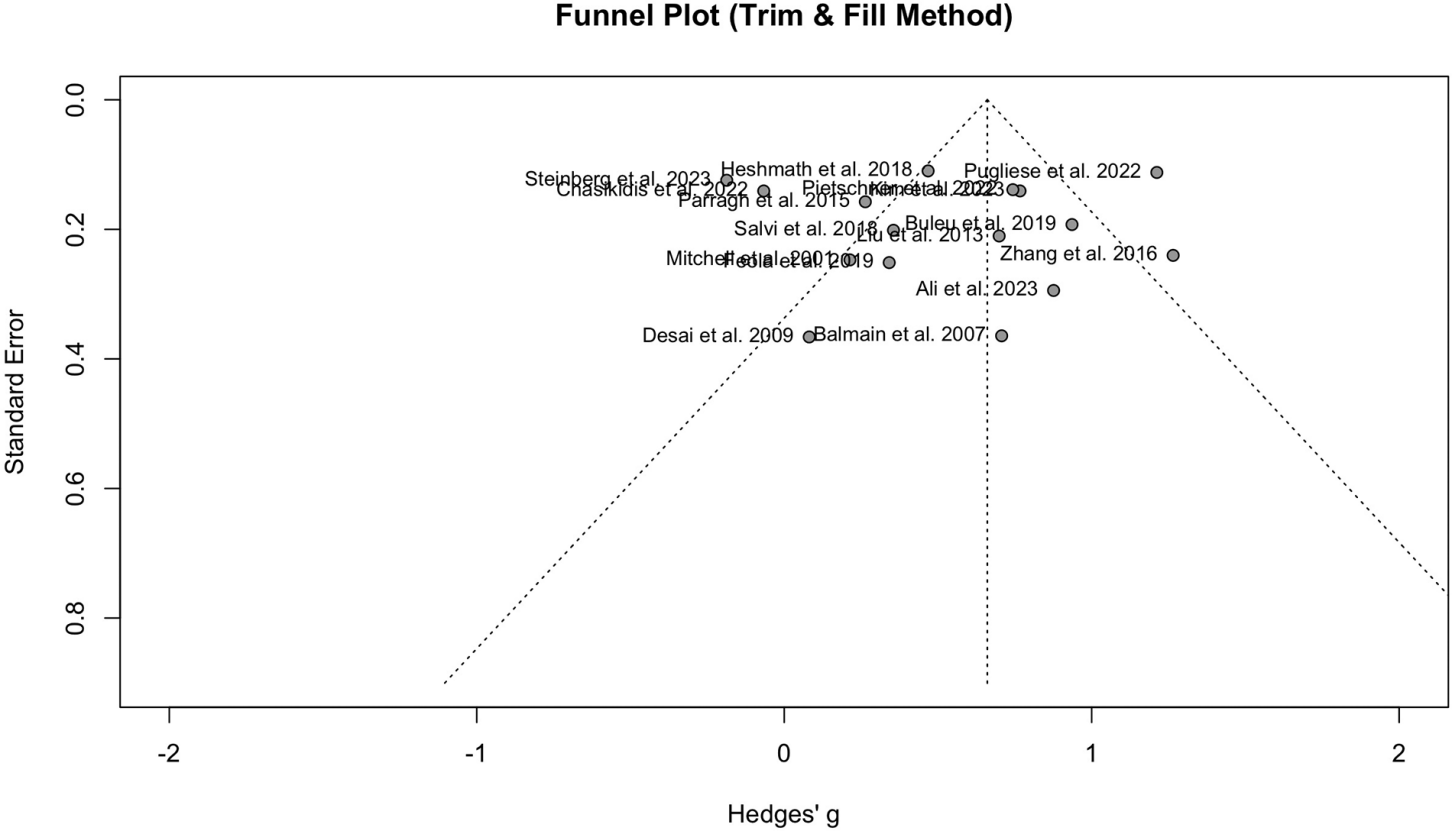
Funnel plot showing the presence of publication bias in the analysis of HF vs. normal population.

Publication bias was detected when comparing PWV between HF and the normal population, according to the funnel plot asymmetry and Egger's test (*P* = 0.04). The funnel plot can be observed in Figure 4.

### 3.4 PWV in HFrEF vs. HFpEF

**Figure 5:**
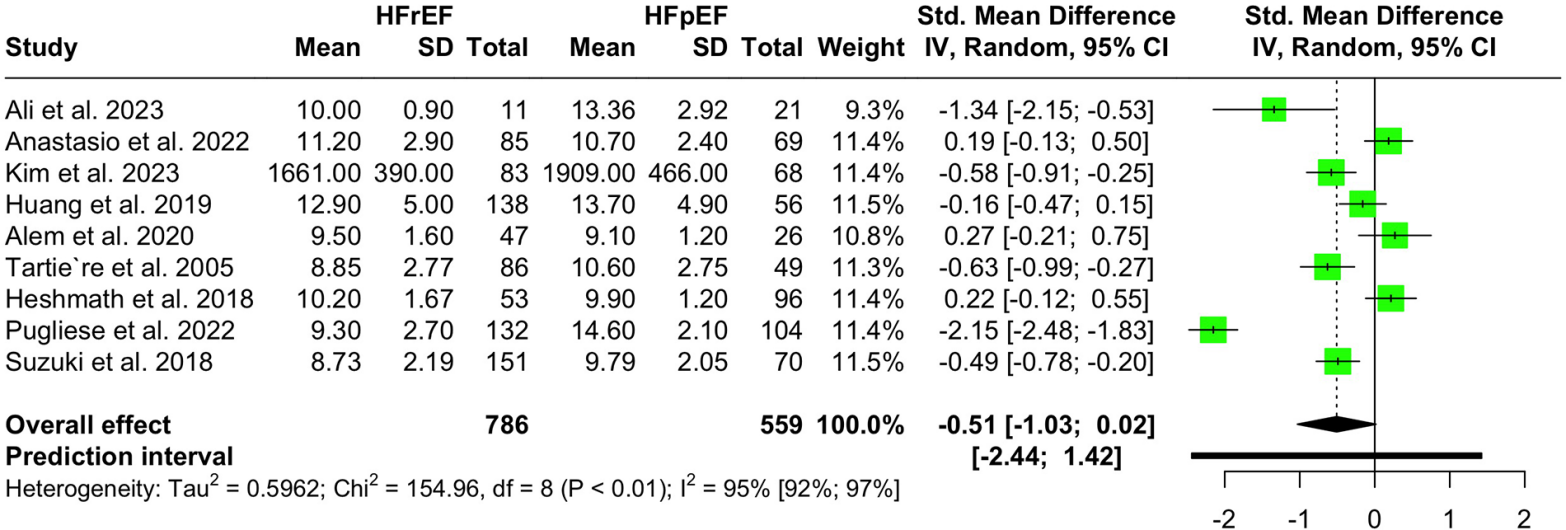
Forest plot showing the PWV difference in hFrEF vs. HFpEF patients.

**Figure 6:**
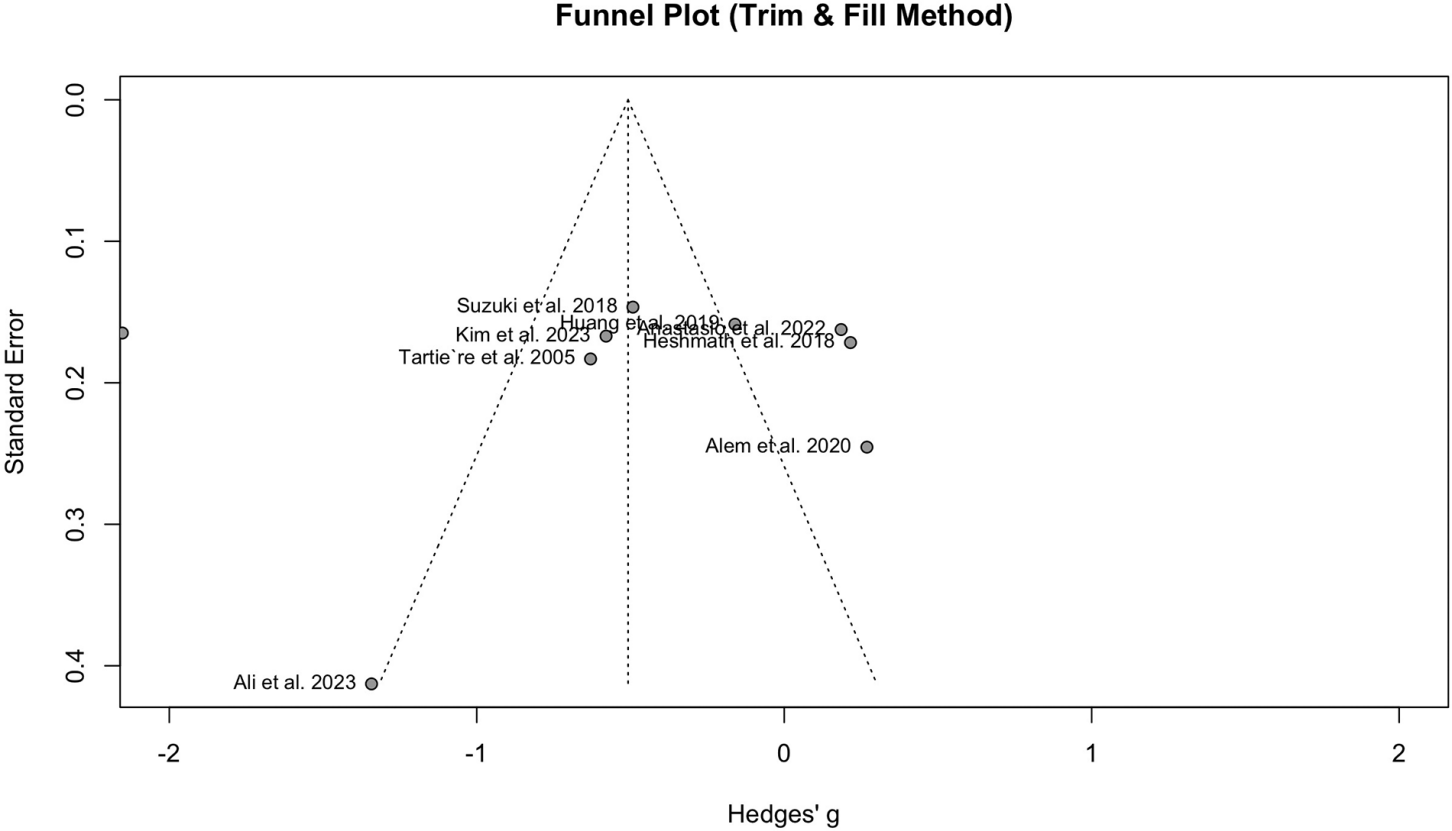
Funnel plot showing no publication bias in the analysis of hFrEF vs. HFpEF patients.

**Figure 7:**
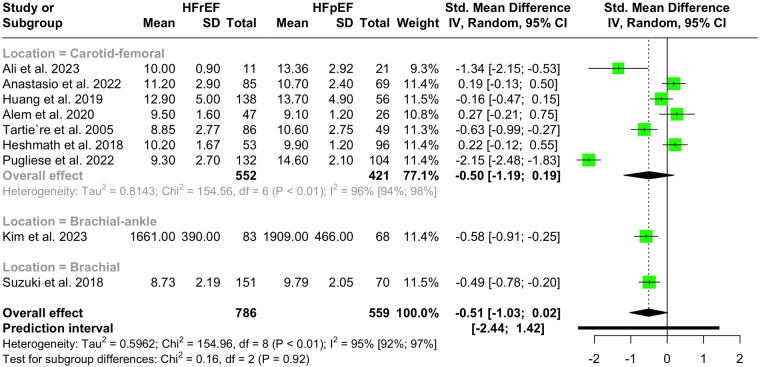
Subgroup analysis of PWV in hFrEF vs. HFpEF patients based on the location of PWV measurement.

Nine studies with a population of 1,345 participants measured PWV among HFrEF and HFpEF patients. As illustrated in Figure 5, our meta-analysis found only a marginally lower PWV in HFrEF patients, which was not statistically significant (SMD −0.51, 95% CI −1.03 to 0.02, *P *= 0.057, *I*^2^ = 95%). Moreover, no change was observed by removing the identified outlier (65) (SMD −0.27, 95% CI −0.60 to 0.05, *P* = 0.1, *I*^2^ = 81%) (Supplementary Figure S8). The funnel plot shows a symmetrical pattern, and Egger's test did not disclose any publication bias (*P* = 0.92) (Figure 6). Subgroup analysis according to the ankle-brachial and brachial measurements of PWV found significantly higher values in HFpEF compared to HFrEF patients (SMD −0.58, 95% CI −0.91 to −0.25; SMD −0.49, 95% CI −0.78 to −0.2) (Figure 7). Findings of the subgroup analysis based on the devices used for the PWV measurement are shown in Supplementary Figure S9. The results show a significantly lower PWV in HFrEF patients compared to HFpEF in the subgroup of studies using non-SphygmoCor devices (SMD −0.41, 95% CI −0.66 to −0.16).

Sensitivity analysis yielded significantly reduced pooled SMD by removing three studies, Alem et al. (45), Heshmath et al. (61), and Anastasio et al. (34) (SMD −0.6, 95% CI −1.16 to −0.05; SMD −0.6, 95% CI −1.16 to −0.04; SMD −0.6, 95% CI −1.16 to −0.04; respectively). (Supplementary Figure S10) Meta-regression showed no statistically significant correlation between any investigated moderators and PWV in the two groups (Supplementary Table S4 and Figures S11-S14).

### 3.5 PWV and adverse outcomes in HF

Thirteen studies have investigated the role of PWV measurements on adverse outcomes in HF patients.

#### 3.5.1 Mortality

Four studies (50, 51, 55, 59) assessed the relationship between PWV and mortality in HF patients. PWV was associated with higher rates of mortality in the study by Demir et al. (50) (OR 1.2, 95% CI 1.04–1.38), Giannitsi et al. (51) (HR 1.32, 95% CI 1.15–1.53), and Regnault et al. (59) (HR 1.16, 95% CI 1.03–1.30). However, Takae et al. (55) found no significant association between mortality and PWV in the HF population.

#### 3.5.2 Mortality and hospitalization

Four studies have explored the correlation of PWV with the composite outcome of death or hospitalization. PWV was associated with higher rates of death or hospitalization reported by Spronck et al. (26) (HR 1.58, 95% CI 1.06–2.36), Bonapace et al. (27) (HR 2.49, 95% CI 1.3–4.6), and Giannitsi et al. (51) (HR 1.12, 95% CI 1.02–1.22). However, no significant association was observed in Dohaei et al.'s investigation (22).

#### 3.5.3 Readmission

The association of PWV with readmission was investigated in two studies (37, 58). PWV was a significant predictor of readmission in the study conducted by Fantin et al. (37) (OR 1.9, 95% CI 1.11–3.44, *P* = 0.02) and Meguro et al. (58) (HR 5.1, 95% CI 1.034–25.166).

#### 3.5.4 Other outcomes

There was a positive correlation between higher levels of PWV (≥1800cm/s) in HFrEF patients and total cardiovascular events (death, hospitalization, nonfatal MI or stroke, unstable angina, and coronary revascularization) in the study by Takae et al. (55) (HR 6.64, 95% CI 1.66 to 26.4). However, Tokitsu et al.'s (56) investigation on HFpEF patients showed a significant association of total cardiovascular events with both the lowest (<1,300 cm/s) and highest (≥2,200 cm/s) quintiles of PWV (HR 2.88, 95% CI 1.12–7.38; HR 2.56, 95% CI 1.28–5.14; respectively). Two studies conducted by Sung et al. found PWV as a predictor of adverse outcomes, including mortality, rehospitalization, nonfatal MI, and stroke within six months (53) and two years (54) following discharge (HR 1.43, 95% CI 1.02–2.00; HR 1.43, 95% CI 1.12–1.82; respectively). In another study, Anastasio et al. (34) reported that PWV was an independent factor of free-event survival in HF patients with acute decompensation (HR 1.7, 95% CI 1.1–2.7).

### 3.6 PWV in specific populations

#### 3.6.1 High cardiovascular risk

Aisu et al. (71) and Lee et al. (72) investigated PWV in patients with cardiovascular risk factors (hypertension, obesity, type 2 diabetes mellitus, atrial fibrillation, and ischemic heart disease). Their results show that higher PWV was associated with hospitalization for new-onset HF (brachial-ankle pulse wave velocity (baPWV) (71): HR 1.28, 95% CI 1.04–1.58; carotid-femoral pulse wave velocity (cfPWV) (72): HR 1.29, 95% CI 1.02–1.63).

#### 3.6.2 Dyspneic patients

Two studies have explored PWV in patients with dyspnea without a diagnosis of HF compared to dyspneic HF patients. Cong et al. (73) assessed baPWV in a cohort of patients with acute dyspnea and found that HF patients presented a higher level of baPWV compared to dyspneic patients without HF diagnosis (OR 2.26, 95% CI 1.15–4.44). The same results were obtained by Weber et al. (78) (OR 1.57, 95% CI 1.28–1.93). These studies suggest using PWV as a predictor of HF in symptomatic patients.

### 3.7 Increased risk of incident HF with a higher PWV

There was a link between higher PWV and the risk of new-onset HF in three studies by Zheng et al. (74) (HR 2.24, 95% CI 1.49–3.38), Heffernan et al. (76) (HR 4.79, 95% CI 2.43–9.45), and Tsao et al. (77) (HR 1.29, 95% CI 1.02–1.64).

## 4 Discussion

### 4.1 Main findings

The systematic review and meta-analysis aimed to assess arterial stiffness and PWV in patients with HF and uncovered various vital discoveries. First, the PWV was notably elevated in individuals with HF compared to the control population, mainly when measured in the carotid-femoral and brachial-ankle regions. However, the disparity in PWV between HFrEF and HFpEF was not statistically significant, especially in the carotid-femoral area. Significantly, elevated PWV levels were associated with higher chances of experiencing various cardiovascular events and adverse outcomes, such as death, hospitalization, and readmission. PWV demonstrated the potential to predict new cases of HF in patients experiencing dyspnea, indicating its potential value in detecting HF in symptomatic patients. Moreover, increased PWV was linked to a heightened likelihood of developing new-onset HF in individuals with cardiovascular risk factors, underscoring its importance as a prognostic indicator for HF onset. These results highlight how PWV is crucial as a predictive marker and could be valuable in evaluating cardiovascular risk and treating patients with HF.

Arterial stiffness is a predictor of cardiovascular events and all-cause mortality (15). Since LV diastolic function is also a predictor of increased mortality from HF (79, 80), increased arterial stiffness may contribute to cardiovascular events by causing diastolic dysfunction. The pathophysiology of HFpEF is characterized by diastolic dysfunction, resulting in insufficient ventricular filling during diastole due to impaired ventricular relaxation and increased stiffness. Even though the LV cavity size is usually normal, the LV wall is thick and stiff, leading to a higher LV mass to end-diastolic volume ratio (81). Increased arterial stiffness plays a crucial role in HFpEF, contributing to higher LV pressure afterload. This compromises ventricular-arterial coordination and exacerbates diastolic dysfunction. The rigidity of large elastic arteries decreases their capacity to absorb the rhythmic flow produced by the heart, resulting in higher LV filling pressures and lower aortic pressures in the relaxation phase, intensifying the pulse pressure (82). The rise in pulse pressure heightens the demand for oxygen in the heart muscle during contraction, leading to LV thickening, further hindering blood flow to the coronary arteries during relaxation (83).

In contrast, HFrEF is defined by systolic dysfunction, where the heart muscle's weakened ability to contract leads to a lower ejection fraction and diminished cardiac output. This can result from a heart attack, inflammation of the heart muscle, or other heart conditions causing changes and expansion of the ventricles (84). In HFrEF, arterial stiffness plays a crucial role in the progression of the disease by exacerbating LV systolic dysfunction (85). Increased vascular resistance and reduced compliance, demonstrated by elevated PWV, lead to a compromised oxygen supply-demand balance in the heart, contributing to adverse changes in cardiac structure. The connection between arterial stiffness and diastolic dysfunction is not solely due to the development of LV hypertrophy, as arterial stiffness is also linked to diastolic dysfunction even when ventricular hypertrophy is present (86, 87).

Arterial tonometry is a simple, non-invasive method to assess arterial rigidity that can be conducted at the patient's bedside (88). Various techniques to measure arterial stiffness include carotid-femoral pulse wave velocity (cfPWV), brachial-ankle pulse wave velocity (baPWV), cardio-ankle vascular index (CAVI), and augmentation index (AIx) (89–91). The baPWV is obtained by dividing the distance between the arms and ankles, as determined by anthropometric data depending on a person's height, by the propagation time of the pulse wave between these two points, which is measured using occlusion cuffs (92). The cfPWV is calculated by manually measuring the distance between the carotid and femoral arteries, then dividing by the time it takes for the pulse wave to travel between the two locations (93). The varying influence of PWV measurement sites on outcomes highlights the significance of considering the specific vascular region under evaluation. The carotid-femoral location is commonly seen as the best method for evaluating central arterial stiffness, offering information on the stiffness of major elastic arteries such as the aorta, which are important for dampening pulsatile flow (94). However, baPWV consistently demonstrates a 17%–20% increase compared to cfPWV (95), showing that baPWV assesses further elements of arterial stiffness. cfPWV focuses solely on the central arterial tree, providing insight into central arterial stiffness, which predominantly affects afterload. In contrast, baPWV considers both the central and peripheral arterial trees, providing a more comprehensive understanding of the afterload effects on diastolic dysfunction by reflecting the overall resistance and compliance of the arterial system. This comprehensive measurement may explain the different values obtained by cfPWV and baPWV. While central arteries primarily determine afterload, peripheral arteries become significant, especially when peripheral arterial disease is present (9). Additional factors that could account for the significant variability in this research may stem from the increased reliance on the person administering the test for cfPWV compared to baPWV, as well as the required use of a handheld pressure transducer to measure pulse waves at the neck and groin (96). Also, the lack of significant differences in PWV between HFrEF and HFpEF, particularly in the carotid-femoral location, may suggest that systemic arterial stiffness is a shared characteristic across both HF phenotypes. A similar systematic meta-analysis was conducted to study the association between arterial stiffness assessed by arterial tonometry and echocardiographic markers of diastolic dysfunction, which is essential for diagnosing heart failure with preserved ejection fraction (HFpEF). Twenty-seven studies included 6,626 patients. baPWV showed significant correlations with the E/A ratio, e0, and E/e0 ratio. Similarly, cfPWV was significantly correlated with the E/A and the E/e0 ratios, but not e0. AIx showed a strong relationship with E/A ratio (*r* = −0.356, 95% CI −0.255 to −0.450), e0 (*r* = −0.313, 95% CI −0.195 to −0.423), and E/e0 ratio (*r* = 0.321, 95% CI 0.250–0.388). CAVI had a strong correlation with the E/A ratio, e0, and baPWV showed a significant correlation with diastolic dysfunction compared to other tonometry techniques (97).

## 5 Strengths and limitations

Adding a significant number of research studies involving 64,687 patients increased the statistical strength and reliability of the meta-analysis. A significant percentage of the studies analyzed were rated as high quality based on their Newcastle-Ottawa Scale (NOS) scores. This enhances the credibility and accuracy of the results. Subgroup analyses were conducted to investigate possible reasons for diversity, like different locations for PWV measurement and HF subtype (HFpEF vs. HFrEF), as well as the devices used for the PWV measurement, leading to a more detailed understanding of the findings. Meta-regression analysis was used to examine how gender, age, and sample size affect observed associations, improving comprehension of potential moderators.

This study carries some limitations. Although attempts were made to deal with differences through subgroup analyses and sensitivity analyses, numerous meta-analyses still showed significant heterogeneity, which may restrict the applicability of the results. Differences in research structure, patient traits, and methods, like variations in PWV measurement methods and anatomical locations, HF subtype (HFpEF vs. HFrEF), and disease severity, probably played a part in the diversity. Also, we could not analyze PWV based on the blood pressure levels, as this information was not sufficiently reported in the included studies. Future research should aim to collect and report BP data to allow for a more comprehensive analysis. Even though the majority of the studies analyzed were well-conducted, a few had methodological flaws that may have influenced the results of the meta-analysis.

## 6 Conclusion

In conclusion, our systematic review and meta-analysis revealed that patients with HF exhibit significantly higher arterial stiffness, as indicated by PWV, compared to the normal population. This association was consistent across various anatomical sites of PWV measurement, including carotid-femoral and ankle-brachial arteries. Furthermore, PWV was found to be a predictor of adverse outcomes in HF patients, including mortality, hospitalization, and readmission. Although there was no statistically significant difference in PWV between patients with HFrEF and HFpEF, subgroup analyses indicated potential differences based on the site of PWV measurement. Additionally, PWV was associated with adverse outcomes in high-risk cardiovascular populations and individuals with dyspnea, underscoring its potential utility as a predictive tool for HF development. Future research should focus on elucidating the underlying mechanisms linking PWV to adverse outcomes in HF patients and exploring its role in risk stratification and therapeutic interventions to improve patient outcomes.

## Data Availability

The original contributions presented in the study are included in the article/**Supplementary Material**, further inquiries can be directed to the corresponding author.
