# Supplementary material for: The association between pulse wave velocity and heart failure: a systematic review and meta-analysis
Source: Front Cardiovasc Med. 2024 Jul 23;11:1435677. doi: 10.3389/fcvm.2024.1435677 (PMC11301612; doi:10.3389/fcvm.2024.1435677)
Supplement: Supplementary file 1 [file Datasheet1.pdf]

Supplementary tables:

Table S1. Search strategy for each database

Query		Results (No.) (August 31, 2023)
**PubMed**		
#1	"Pulse Wave Analysis" OR "Carotid-Femoral Pulse Wave Velocity" OR "Carotid-Femoral Pulse Wave Velocit*" OR "Ankle-Brachial Pulse Wave Velocit*" OR "Pulse Transit Time" OR "Pulse Wave Transit Time" OR "Pulse Wave Velocity" OR "pulse wave analys*"	14,131
#2	"Heart Failure, Systolic" OR "Heart Failure" OR "Heart Failure, Diastolic" OR “Heart Failure” OR “Cardiac Failure” OR “Congestive Heart Failure” OR “Heart Decompensation” OR “Left-Sided Heart Failure” OR “Right-Sided Heart Failure” OR “Myocardial Failure” OR “Systolic Heart Failure” OR “Systolic dysfunction” OR “diastolic dysfunction” OR “Heart Failure, Reduced Ejection Fraction” OR “Heart Failure with Reduced Ejection Fraction” OR “Diastolic Heart Failure” OR “Heart Failure, Normal Ejection Fraction” OR “Heart Failure with Normal Ejection Fraction” OR “Heart Failure, Preserved Ejection Fraction” OR “Heart Failure with Preserved Ejection Fraction”	280,657
#3	#1 AND #2	624
**Embase**		
#1	"Pulse Wave Analysis" OR "Carotid-Femoral Pulse Wave Velocity" OR "Carotid-Femoral Pulse Wave Velocit*" OR "Ankle-Brachial Pulse Wave Velocit*" OR "Pulse Transit Time" OR "Pulse Wave Transit Time" OR "Pulse Wave Velocity" OR "pulse wave analys*"	35,476
#2	"Heart Failure, Systolic" OR "Heart Failure" OR "Heart Failure, Diastolic" OR “Heart Failure” OR “Cardiac Failure” OR “Congestive Heart Failure” OR “Heart Decompensation” OR “Left-Sided Heart Failure” OR “Right-Sided Heart Failure” OR “Myocardial Failure” OR “Systolic Heart Failure” OR “Systolic dysfunction” OR “diastolic dysfunction” OR “Heart Failure, Reduced Ejection Fraction” OR “Heart Failure with Reduced Ejection Fraction” OR “Diastolic Heart Failure” OR “Heart Failure, Normal Ejection Fraction” OR “Heart Failure with Normal Ejection Fraction” OR “Heart Failure, Preserved Ejection Fraction” OR “Heart Failure with Preserved Ejection Fraction”	741,671
#3	#1 AND #2	3,274
**Web of Science**		
#1	TS= ("Pulse Wave Analysis" OR "Carotid-Femoral Pulse Wave Velocity" OR "Carotid-Femoral Pulse Wave Velocit*" OR "Ankle-Brachial Pulse Wave Velocit*" OR "Pulse Transit Time" OR "Pulse Wave Transit Time" OR "Pulse Wave Velocity" OR "pulse wave analys*")	16,688
#2	TS=("Heart Failure, Systolic" OR "Heart Failure" OR "Heart Failure, Diastolic" OR “Heart Failure” OR “Cardiac Failure” OR “Congestive Heart Failure” OR “Heart Decompensation” OR “Left-Sided Heart Failure” OR	319,028

	“Right-Sided Heart Failure” OR “Myocardial Failure” OR “Systolic Heart Failure” OR “Systolic dysfunction” OR “diastolic dysfunction” OR “Heart Failure, Reduced Ejection Fraction” OR “Heart Failure with Reduced Ejection Fraction” OR “Diastolic Heart Failure” OR “Heart Failure, Normal Ejection Fraction” OR “Heart Failure with Normal Ejection Fraction” OR “Heart Failure, Preserved Ejection Fraction” OR “Heart Failure with Preserved Ejection Fraction”)	
#3	#1 AND #2	1,086
**SCOPUS**		
#1	TITLE-ABS-KEY("Pulse Wave Analysis" OR "Carotid-Femoral Pulse Wave Velocity" OR "Carotid-Femoral Pulse Wave Velocit*" OR "Ankle-Brachial Pulse Wave Velocit*" OR "Pulse Transit Time" OR "Pulse Wave Transit Time" OR "Pulse Wave Velocity" OR "pulse wave analys*")	17,543
#2	TITLE-ABS-KEY("Heart Failure, Systolic" OR "Heart Failure" OR "Heart Failure, Diastolic" OR “Heart Failure” OR “Cardiac Failure” OR “Congestive Heart Failure” OR “Heart Decompensation” OR “Left-Sided Heart Failure” OR “Right-Sided Heart Failure” OR “Myocardial Failure” OR “Systolic Heart Failure” OR “Systolic dysfunction” OR “diastolic dysfunction” OR “Heart Failure, Reduced Ejection Fraction” OR “Heart Failure with Reduced Ejection Fraction” OR “Diastolic Heart Failure” OR “Heart Failure, Normal Ejection Fraction” OR “Heart Failure with Normal Ejection Fraction” OR “Heart Failure, Preserved Ejection Fraction” OR “Heart Failure with Preserved Ejection Fraction”)	423,118
#3	#1 AND #2	993
TOTAL RECORDS		***5977***
TOTAL RECORDS AFTER REMOVING DUPLICATES		***2974***

Table S2. Qualities of the included studies based on NOS

Study	Year	Selection				Comparability	Outcome			Overall score	Overall quality
Abolfazl Dohaei	2017	*	-	*	*	*	*	-	-	5	Satisfactory
Ahmed El Fol	2022	*	-	*	*	**	*	*	*	8	Good
ASHISH SHAH	2009	*	*	*	*	*	*	-	*	7	Good
Sean Balmain	2007	*	*	*	*	*	*	-	*	7	Good
Bart Spronck	2021	*	*	*	*	**	*	*	*	9	Very Good
Stefano Bonapace	2013	*	*	*	*	*	*	*	-	7	Good
Florina N. Buleu	2019	*	*	*	*	**	*	*	*	9	Very Good
Christos Chasikidis	2022	*	*	*	*	**	*	*	*	9	Very Good
Chengyu Liu	2013	*	*	-	*	*	*	*	*	7	Good
Metin Coksevim	2020	*	*	*	*	**	-	*	*	8	Good
Danish Ali	2023	*	*	-	*	**	*	*	*	8	Good
AKSHAY S. DESAI	2009	*	*	*	*	*	-	*	*	7	Good
Fabio Anastasio	2022	*	*	*	*	**	*	-	*	8	Good
Lajos Fehérvári (evaluation	2021	*	*	*	*	*	*	-	-	6	Satisfactory
Lajos Fehérvári (serum)	2021	*	*	*	*	**	*	*	-	8	Good
Mauro Feola	2019	*	*	*	*	**	*	*	-	8	Good

Francesco Fantin	2022	*	*	*	*	**	*	*	*	9	Very Good
Hack-Lyoung Kim	2023	*	*	*	*	**	*	-	*	8	Good
Po-Chao Hsu	2010	*	*	*	-	*	*	-	*	6	Satisfactory
Wei-Ming Huang	2019	*	*	*	*	**	*	*	*	9	Very Good
El-Sayed H. Ibrahim	2011	*	-	*	*	*	*	*	*	7	Good
J. Malcolm 0. Arnold,	1991	*	-	*	*	*	*	-	*	6	Satisfactory
Jing Zhang	2016	*	*	-	*	*	-	-	*	5	Satisfactory
Dong-Bin Kim	2013	*	-	*	*	*	*	*	*	7	Good
Manal M. Alem	2020	-	*	*	*	*	*	*	-	6	Satisfactory
Gary F. Mitchell	2001	*	*	*	*	*	-	*	*	7	Good
Rebecca S. Steinberg	2023	*	*	*	*	**	*	*	*	9	Very Good
Robert Pietschner	2022	*	*	*	-	**	*	*	*	8	Good
Paolo Salvi	2018	*	*	*	*	*	*	*	-	7	Good
Şerafettin Demir	2013	*	*	*	*	**	*	*	*	9	Very Good
Sophia Giannitsi	2020	*	-	-	*	*	*	*	-	5	Satisfactory
Stephanie Parragh	2019	*	*	*	*	*	-	*	*	7	Good
Shih-Hsien Sung	2011	*	*	*	*	**	*	*	*	9	Very Good
Shih-Hsien Sung	2012	*	*	*	*	**	*	*	*	9	Very Good

Masafumi Takae	2019	*	*	*	*	**	*	*	*	9	Very Good
Takanori Tokitsu	2017	*	*	*	*	**	*	*	*	9	Very Good
J-M Tartie`re	2005	*	*	*	*	*	-	*	*	7	Good
Tomomi Meguro	2009	*	*	*	*	**	*	*	*	9	Very Good
Veronique Regnault	2013	*	*	*	*	**	*	*	*	9	Very Good
Vyacheslav Ryabov	2012	*	-	*	*	*	*	-	-	5	Satisfactory
Zeba Hashmath	2018	*	*	*	*	**	*	*	*	9	Very Good
Stefano Bonapace	2016	*	*	*	*	**	*	*	*	9	Very Good
Daniel R. Kaiser	2001	*	*	*	-	*	*	*	*	7	Good
Min Sun	2023	*	-	*	*	*	-	*	*	6	Satisfactory
Nicola Riccardo Pugliese	2022	*	*	*	*	**	*	*	*	9	Very Good
Alberto Radaelli	2010	*	*	*	*	**	*	*	*	9	Very Good
Alberto Radaelli	2014	*	*	*	*	**	*	*	*	9	Very Good
Satoshi Suzuki	2018	*	*	*	*	*	*	-	*	7	Good
Takanori Tokitsu	2016	*	*	*	*	**	*	*	*	9	Very Good
Nikita Trembach	2018	*	*	-	*	*	*	*	-	6	Satisfactory
Hiroe Aisu	2016	*	*	*	*	**	*	*	*	9	Very Good
Chan Joo Lee	2021	*	*	*	*	*	*	*	*	9	Very Good

Tao Cong	2015	*	-	-	*	*	*	-	*	5	Satisfactory
Hongwei Zheng	2023	*	*	*	*	**	*	*	*	9	Very Good
Sheng Kang	2010	*	*	*	*	**	*	*	*	9	Very Good
Kevin S. Heffernan	2022	*	*	*	*	**	*	*	*	9	Very Good
Connie W. Tsao	2016	*	*	*	*	**	*	*	*	9	Very Good
Thomas Weber	2013	*	*	*	*	**	*	*	*	9	Very Good

Table S3: Meta-regression of PWV in HF patients vs. controls

Moderator	No. of comparisons	No. of subjects		Meta-regression			R^2 Analog (proportion of variance explained) (%)
		HF	Control	Slope	95% CI	P value	
Age	19	1385	1277	0.0030	-0.0967; 0.1026	0.9537	0
Year	19	1385	1277	-0.0637	-0.1337; 0.0064	0.0748	12.07
Sample size	19	1385	1277	-0.0039	-0.0096; 0.0018	0.1843	2.04
Male	17	1309	1226	-0.0166	-0.0315; -0.0017	0.0286	2.76

Table S4: Meta-regression of PWV in HFrEF vs. HfpEF

Moderator	No. of comparisons	No. of subjects		Meta-regression			R^2 Analog (proportion of variance explained) (%)
		HfrEF	HfpEF	Slope	95% CI	P value	

Age	9	786	559	-0.0095	-0.1018; 0.0828	0.8396	0
Year	9	786	559	-0.0173	-0.1227; 0.0882	0.7481	0
Sample size	9	786	559	-0.0031	-0.0120; 0.0058	0.4972	0
Male gender	9	786	559	0.0401	-0.0014; 0.0816	0.0580	24.85

Supplementary figures:

Fig S1:

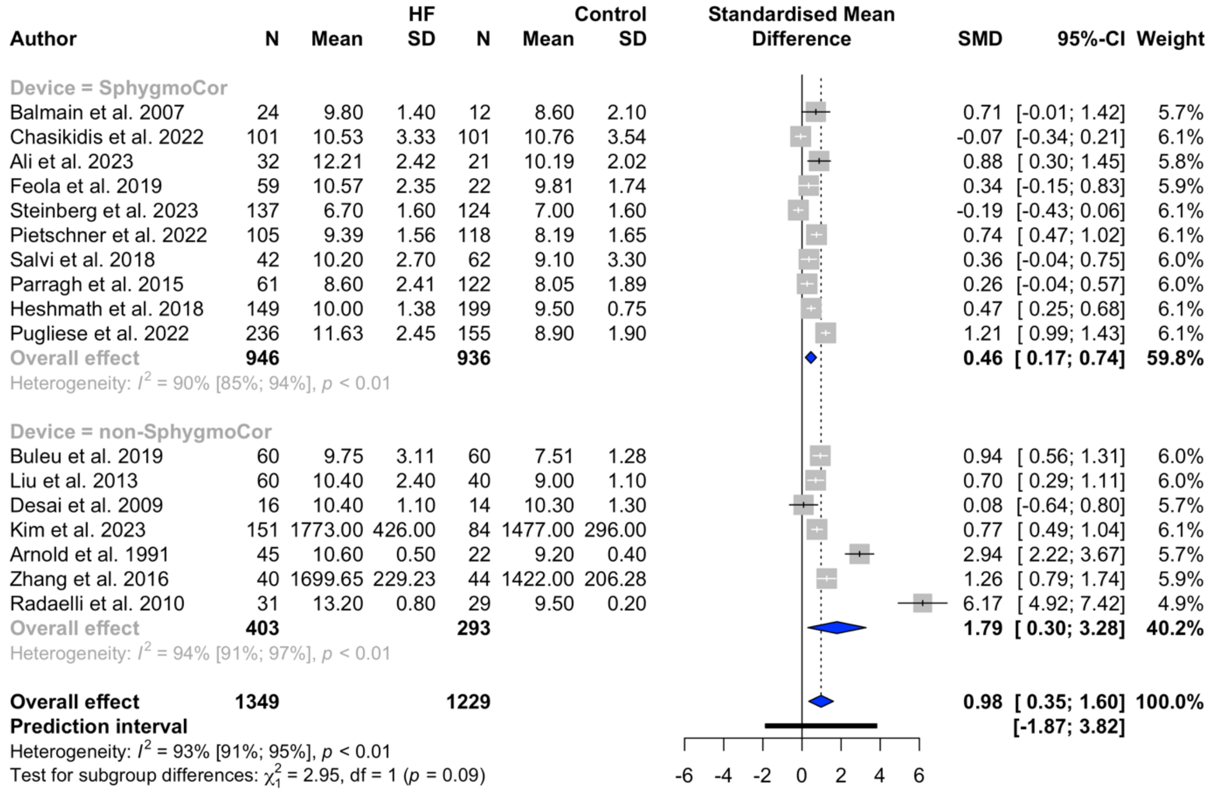

Fig S2:

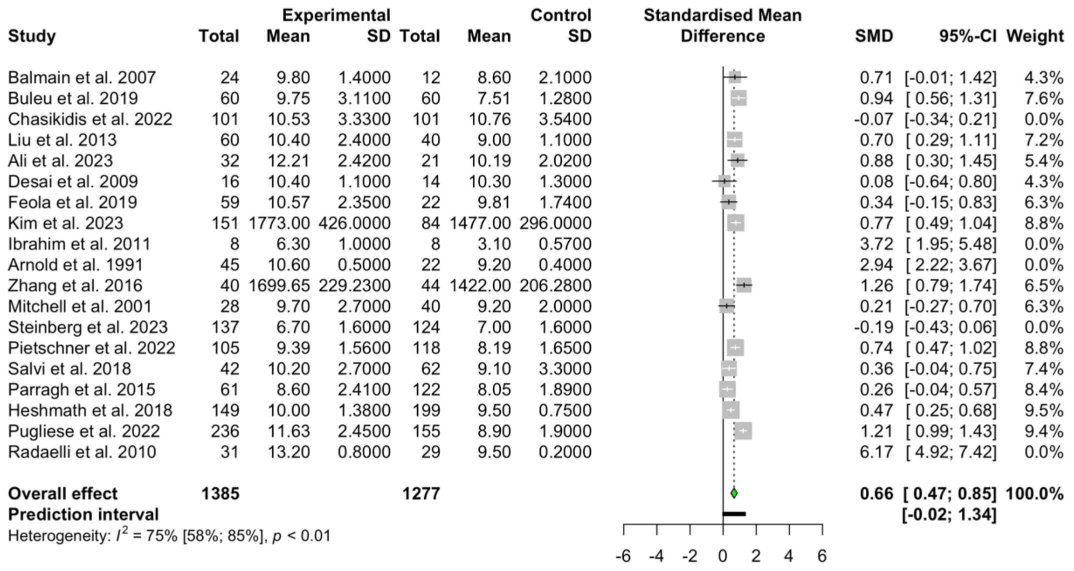
Experimental
Control
Standardised Mean Difference
Study Total Mean SD Total Mean SD SMD 95%-CI Weight
Balmain et al. 2007 24 9.80 1.4000 12 8.60 2.1000 0.71 [-0.01; 1.42] 4.3%
Buleu et al. 2019 60 9.75 3.1100 60 7.51 1.2800 0.94 [0.56; 1.31] 7.6%
Chasikidis et al. 2022 101 10.53 3.3300 101 10.76 3.5400 -0.07 [-0.34; 0.21] 0.0%
Liu et al. 2013 60 10.40 2.4000 40 9.00 1.1000 0.70 [0.29; 1.11] 7.2%
Ali et al. 2023 32 12.21 2.4200 21 10.19 2.0200 0.88 [0.30; 1.45] 5.4%
Desai et al. 2009 16 10.40 1.1000 14 10.30 1.3000 0.08 [-0.64; 0.80] 4.3%
Feola et al. 2019 59 10.57 2.3500 22 9.81 1.7400 0.34 [-0.15; 0.83] 6.3%
Kim et al. 2023 151 1773.00 426.0000 84 1477.00 296.0000 0.77 [0.49; 1.04] 8.8%
Ibrahim et al. 2011 8 6.30 1.0000 8 3.10 0.5700 3.72 [1.95; 5.48] 0.0%
Arnold et al. 1991 45 10.60 0.5000 22 9.20 0.4000 2.94 [2.22; 3.67] 0.0%
Zhang et al. 2016 40 1699.65 229.2300 44 1422.00 206.2800 1.26 [0.79; 1.74] 6.5%
Mitchell et al. 2001 28 9.70 2.7000 40 9.20 2.0000 0.21 [-0.27; 0.70] 6.3%
Steinberg et al. 2023 137 6.70 1.6000 124 7.00 1.6000 -0.19 [-0.43; 0.06] 0.0%
Pietschner et al. 2022 105 9.39 1.5600 118 8.19 1.6500 0.74 [0.47; 1.02] 8.8%
Salvi et al. 2018 42 10.20 2.7000 62 9.10 3.3000 0.36 [-0.04; 0.75] 7.4%
Parragh et al. 2015 61 8.60 2.4100 122 8.05 1.8900 0.26 [-0.04; 0.57] 8.4%
Heshmath et al. 2018 149 10.00 1.3800 199 9.50 0.7500 0.47 [0.25; 0.68] 9.5%
Pugliese et al. 2022 236 11.63 2.4500 155 8.90 1.9000 1.21 [0.99; 1.43] 9.4%
Radaelli et al. 2010 31 13.20 0.8000 29 9.50 0.2000 6.17 [4.92; 7.42] 0.0%
Overall effect 1385 1277 0.66 [0.47; 0.85] 100.0%
Prediction interval [-0.02; 1.34]
Heterogeneity: I2 = 75% [58%; 85%], p < 0.01
-6 -4 -2 0 2 4 6

Fig S3:

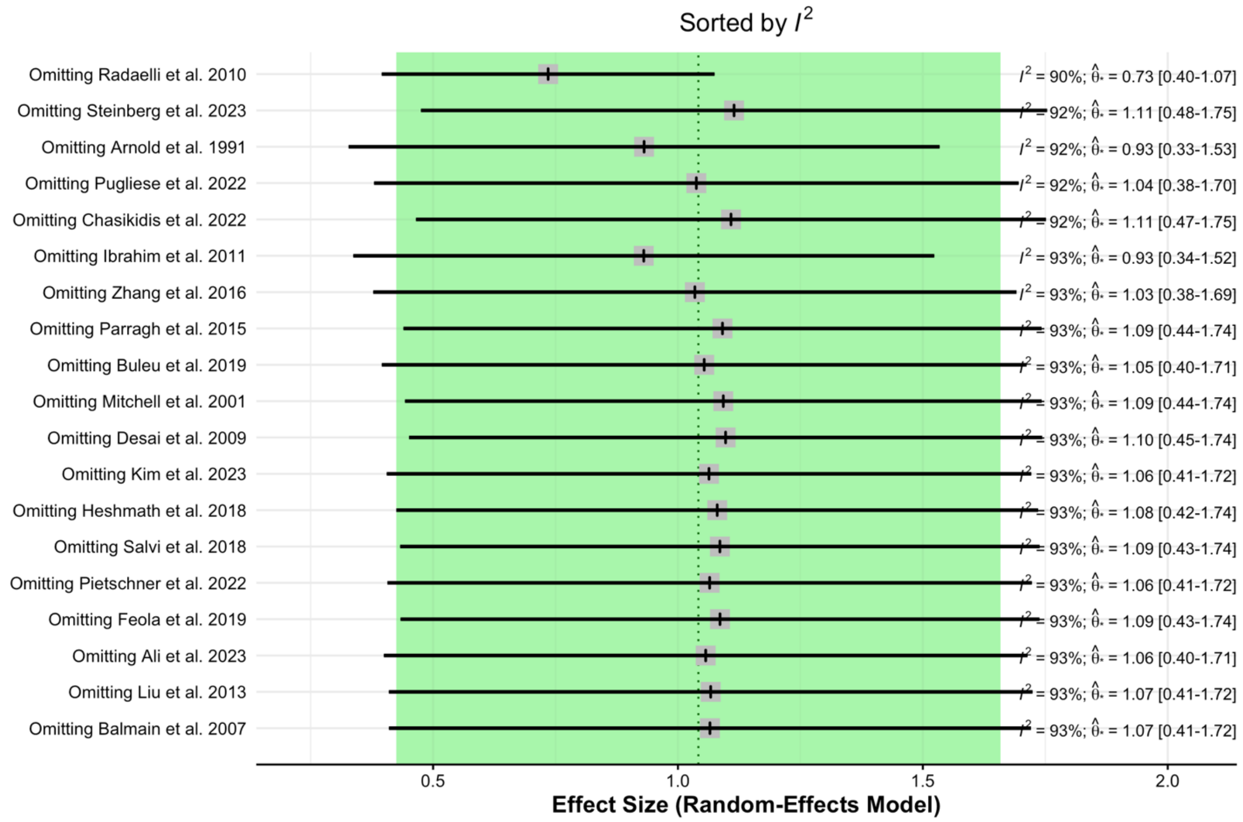
Sorted by I2
Omitting Radaelli et al. 2010 I2 = 90%; θ̂* = 0.73 [0.40-1.07]
Omitting Steinberg et al. 2023 I2 = 92%; θ̂* = 1.11 [0.48-1.75]
Omitting Arnold et al. 1991 I2 = 92%; θ̂* = 0.93 [0.33-1.53]
Omitting Pugliese et al. 2022 I2 = 92%; θ̂* = 1.04 [0.38-1.70]
Omitting Chasikidis et al. 2022 I2 = 92%; θ̂* = 1.11 [0.47-1.75]
Omitting Ibrahim et al. 2011 I2 = 93%; θ̂* = 0.93 [0.34-1.52]
Omitting Zhang et al. 2016 I2 = 93%; θ̂* = 1.03 [0.38-1.69]
Omitting Parragh et al. 2015 I2 = 93%; θ̂* = 1.09 [0.44-1.74]
Omitting Buleu et al. 2019 I2 = 93%; θ̂* = 1.05 [0.40-1.71]
Omitting Mitchell et al. 2001 I2 = 93%; θ̂* = 1.09 [0.44-1.74]
Omitting Desai et al. 2009 I2 = 93%; θ̂* = 1.10 [0.45-1.74]
Omitting Kim et al. 2023 I2 = 93%; θ̂* = 1.06 [0.41-1.72]
Omitting Heshmath et al. 2018 I2 = 93%; θ̂* = 1.08 [0.42-1.74]
Omitting Salvi et al. 2018 I2 = 93%; θ̂* = 1.09 [0.43-1.74]
Omitting Pietschner et al. 2022 I2 = 93%; θ̂* = 1.06 [0.41-1.72]
Omitting Feola et al. 2019 I2 = 93%; θ̂* = 1.09 [0.43-1.74]
Omitting Ali et al. 2023 I2 = 93%; θ̂* = 1.06 [0.40-1.71]
Omitting Liu et al. 2013 I2 = 93%; θ̂* = 1.07 [0.41-1.72]
Omitting Balmain et al. 2007 I2 = 93%; θ̂* = 1.07 [0.41-1.72]
0.5 1.0 1.5 2.0
Effect Size (Random-Effects Model)

Fig S4:

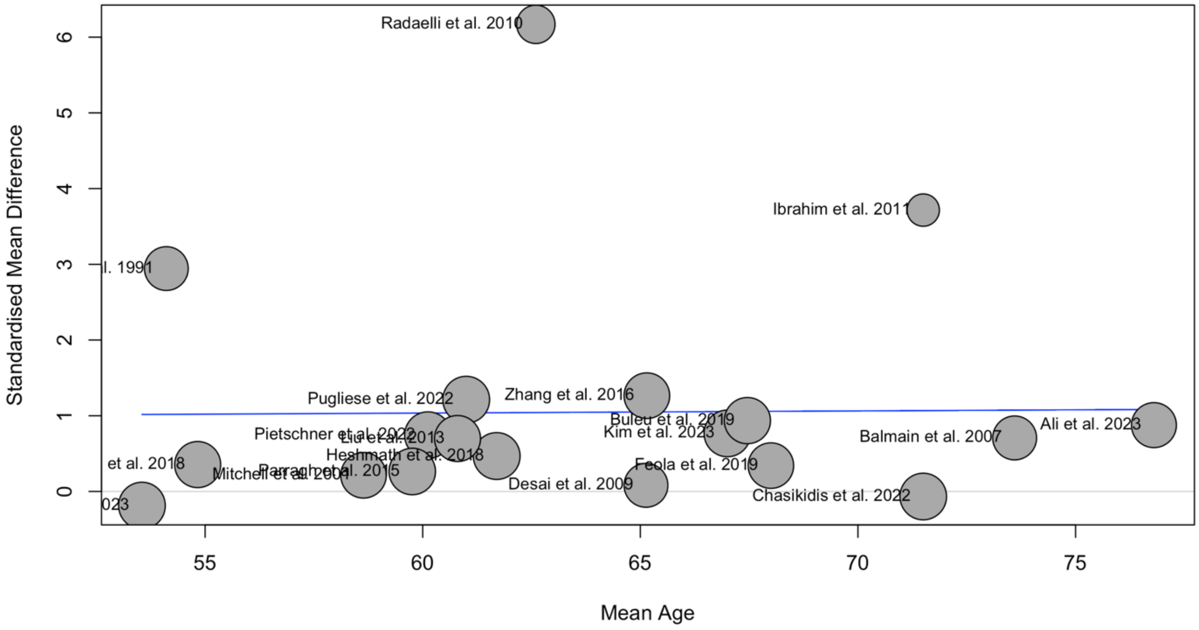
Radaelli et al. 2010
Ibrahim et al. 2011
l. 1991
Pugliese et al. 2022
Zhang et al. 2016
Buleu et al. 2019
Kim et al. 2023
Balmain et al. 2007
Ali et al. 2023
Pietschner et al. 2022
Liu et al. 2013
Heshmath et al. 2018
et al. 2018
Mitchell et al. 2001
Parragh et al. 2015
Desai et al. 2009
Feola et al. 2019
Chasikidis et al. 2022
023
Standardised Mean Difference
Mean Age

Fig S5:

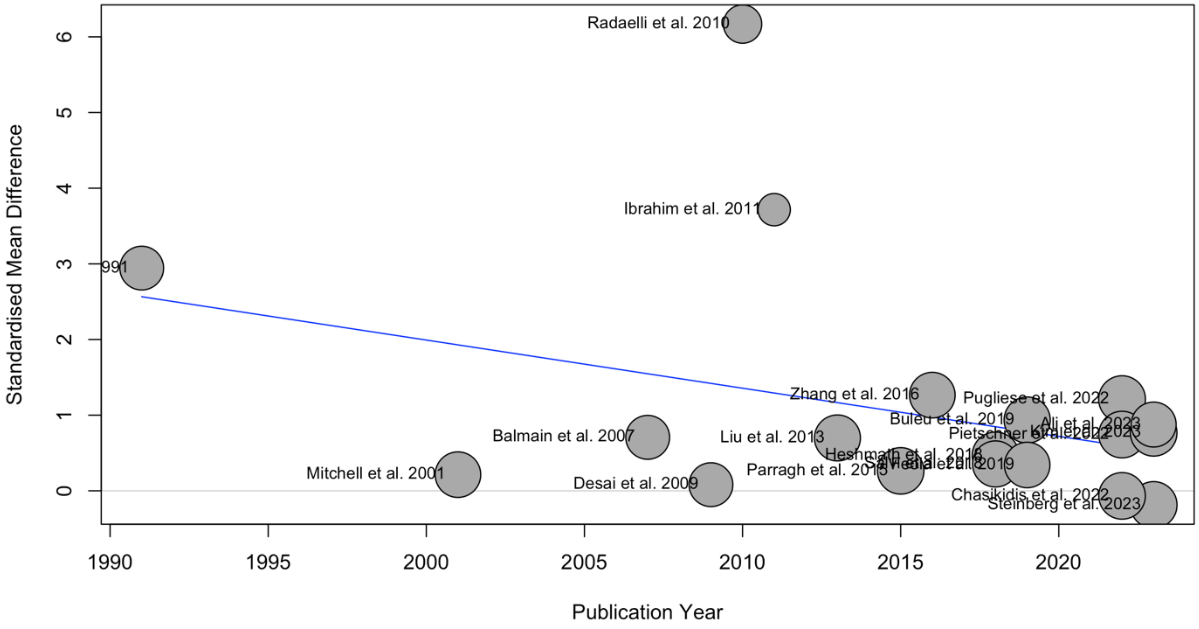
Radaelli et al. 2010
Ibrahim et al. 2011
991
Zhang et al. 2016
Pugliese et al. 2022
Buleu et al. 2019
Ali et al. 2023
Pietschner et al. 2022
Balmain et al. 2007
Liu et al. 2013
Heshmath et al. 2018
Parragh et al. 2015
Mitchell et al. 2001
Desai et al. 2009
Chasikidis et al. 2022
Steinberg et al. 2023
Standardised Mean Difference
Publication Year

Fig S6:

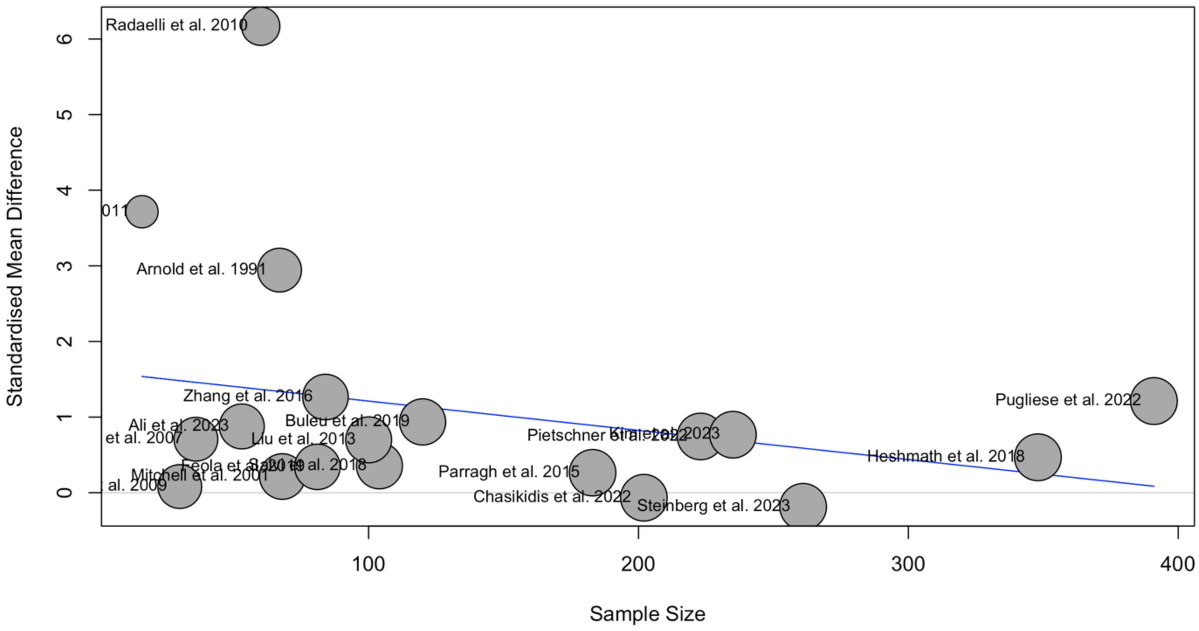
Radaelli et al. 2010
Arnold et al. 1991
Zhang et al. 2016
Ali et al. 2023
Buleu et al. 2019
Liu et al. 2013
Mitchell et al. 2001
Parragh et al. 2015
Chasikidis et al. 2022
Steinberg et al. 2023
Pugliese et al. 2022
Heshmath et al. 2018
Standardised Mean Difference
Sample Size
0
1
2
3
4
5
6
100
200
300
400

Fig S7:

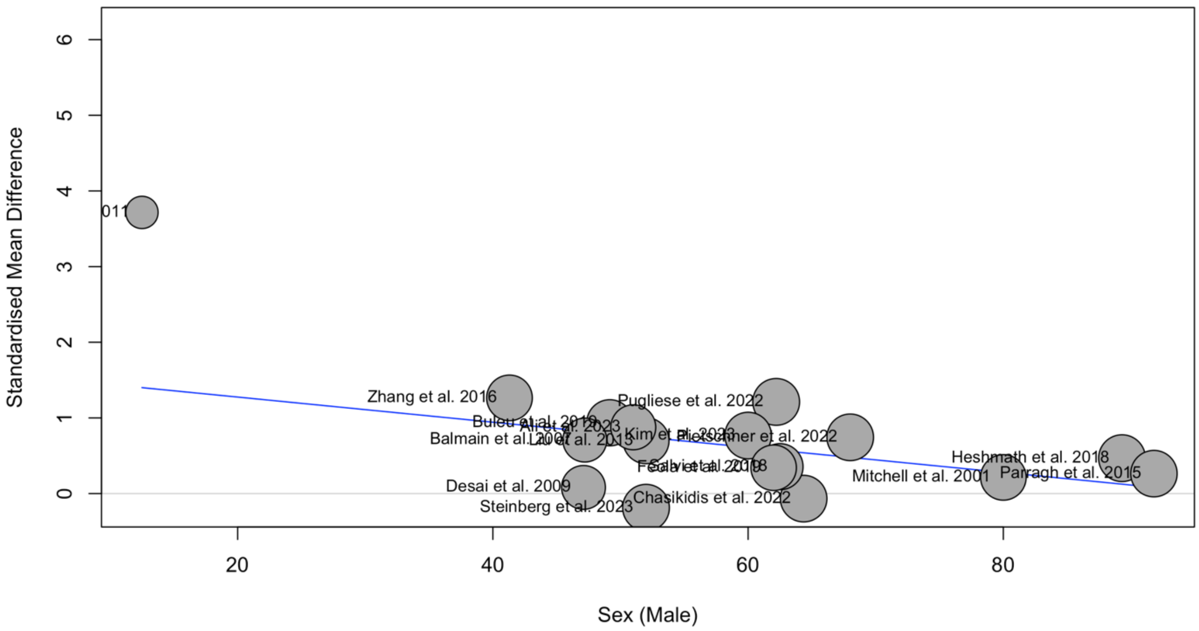
Zhang et al. 2016
Pugliese et al. 2022
Balmain et al. 2007
Desai et al. 2009
Steinberg et al. 2023
Chasikidis et al. 2022
Mitchell et al. 2001
Heshmath et al. 2018
Parragh et al. 2015
Standardised Mean Difference
Sex (Male)
0
1
2
3
4
5
6
20
40
60
80

Fig S8:

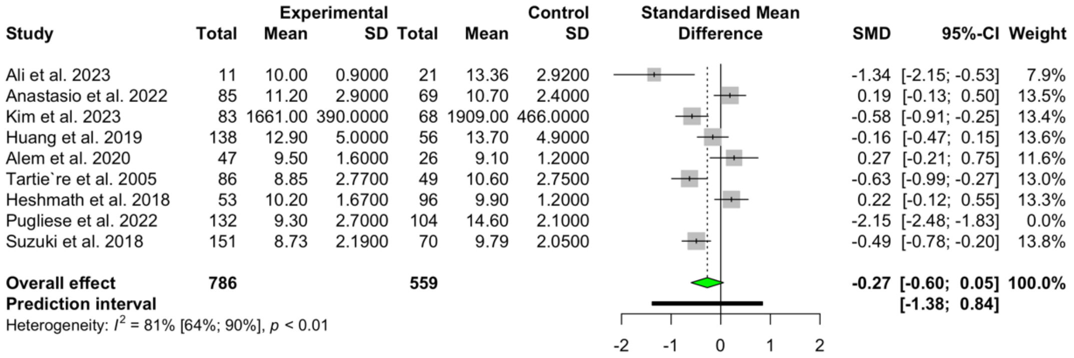

Experimental
Control
Standardised Mean Difference
Study Total Mean SD Total Mean SD
SMD 95%-CI Weight
Ali et al. 2023 11 10.00 0.9000 21 13.36 2.9200 -1.34 [-2.15; -0.53] 7.9%
Anastasio et al. 2022 85 11.20 2.9000 69 10.70 2.4000 0.19 [-0.13; 0.50] 13.5%
Kim et al. 2023 83 1661.00 390.0000 68 1909.00 466.0000 -0.58 [-0.91; -0.25] 13.4%
Huang et al. 2019 138 12.90 5.0000 56 13.70 4.9000 -0.16 [-0.47; 0.15] 13.6%
Alem et al. 2020 47 9.50 1.6000 26 9.10 1.2000 0.27 [-0.21; 0.75] 11.6%
Tartie`re et al. 2005 86 8.85 2.7700 49 10.60 2.7500 -0.63 [-0.99; -0.27] 13.0%
Heshmath et al. 2018 53 10.20 1.6700 96 9.90 1.2000 0.22 [-0.12; 0.55] 13.3%
Pugliese et al. 2022 132 9.30 2.7000 104 14.60 2.1000 -2.15 [-2.48; -1.83] 0.0%
Suzuki et al. 2018 151 8.73 2.1900 70 9.79 2.0500 -0.49 [-0.78; -0.20] 13.8%
Overall effect 786 559 -0.27 [-0.60; 0.05] 100.0%
Prediction interval [-1.38; 0.84]
Heterogeneity: I^2 = 81% [64%; 90%], p < 0.01
-2 -1 0 1 2

Fig S9:

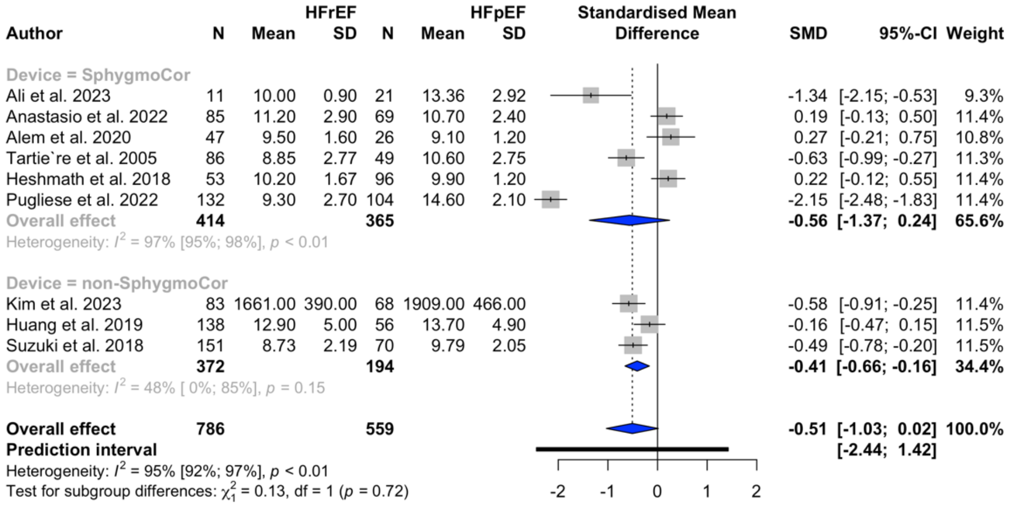

HFrEF
HFpEF
Standardised Mean Difference
Author N Mean SD N Mean SD
SMD 95%-CI Weight
Device = SphygmoCor
Ali et al. 2023 11 10.00 0.90 21 13.36 2.92 -1.34 [-2.15; -0.53] 9.3%
Anastasio et al. 2022 85 11.20 2.90 69 10.70 2.40 0.19 [-0.13; 0.50] 11.4%
Alem et al. 2020 47 9.50 1.60 26 9.10 1.20 0.27 [-0.21; 0.75] 10.8%
Tartie`re et al. 2005 86 8.85 2.77 49 10.60 2.75 -0.63 [-0.99; -0.27] 11.3%
Heshmath et al. 2018 53 10.20 1.67 96 9.90 1.20 0.22 [-0.12; 0.55] 11.4%
Pugliese et al. 2022 132 9.30 2.70 104 14.60 2.10 -2.15 [-2.48; -1.83] 11.4%
Overall effect 414 365 -0.56 [-1.37; 0.24] 65.6%
Heterogeneity: I^2 = 97% [95%; 98%], p < 0.01
Device = non-SphygmoCor
Kim et al. 2023 83 1661.00 390.00 68 1909.00 466.00 -0.58 [-0.91; -0.25] 11.4%
Huang et al. 2019 138 12.90 5.00 56 13.70 4.90 -0.16 [-0.47; 0.15] 11.5%
Suzuki et al. 2018 151 8.73 2.19 70 9.79 2.05 -0.49 [-0.78; -0.20] 11.5%
Overall effect 372 194 -0.41 [-0.66; -0.16] 34.4%
Heterogeneity: I^2 = 48% [0%; 85%], p = 0.15
Overall effect 786 559 -0.51 [-1.03; 0.02] 100.0%
Prediction interval [-2.44; 1.42]
Heterogeneity: I^2 = 95% [92%; 97%], p < 0.01
Test for subgroup differences: χ^2_1 = 0.13, df = 1 (p = 0.72)
-2 -1 0 1 2

Fig S10:

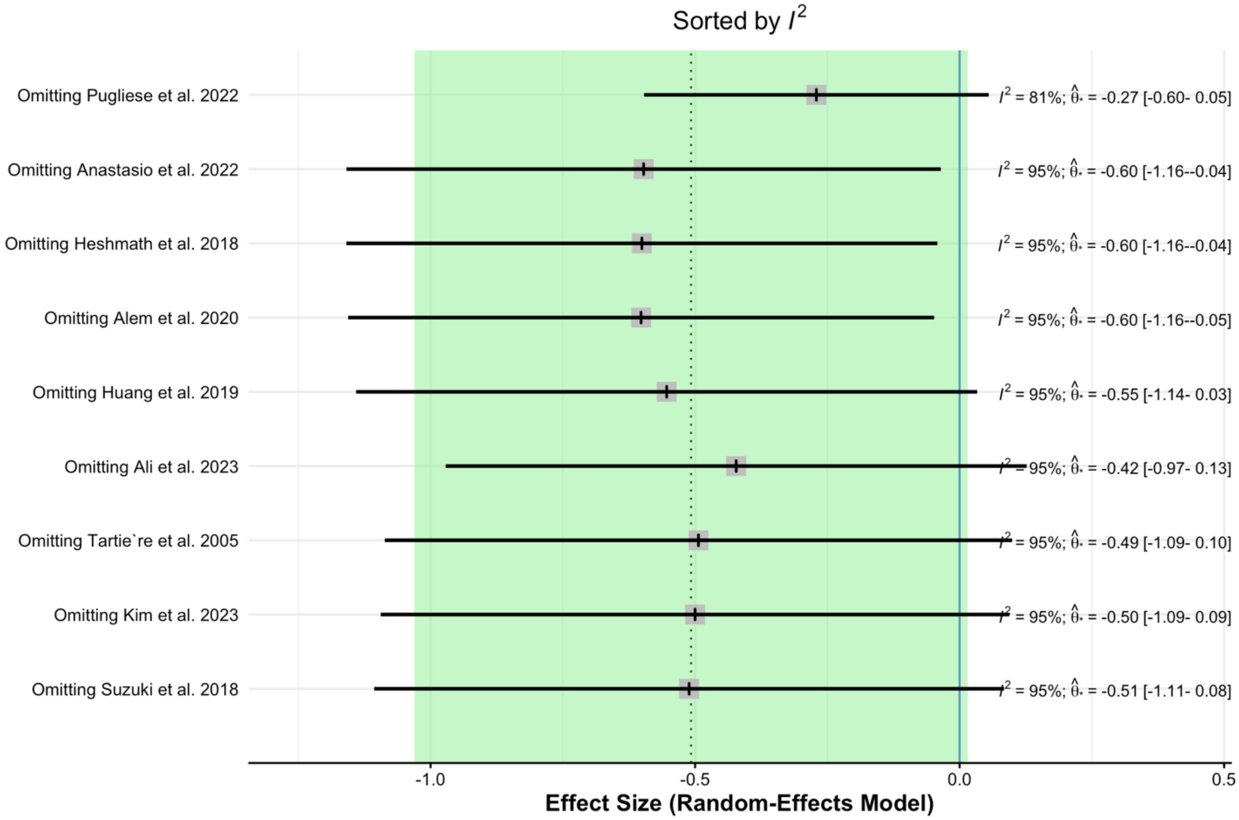

Fig S11:

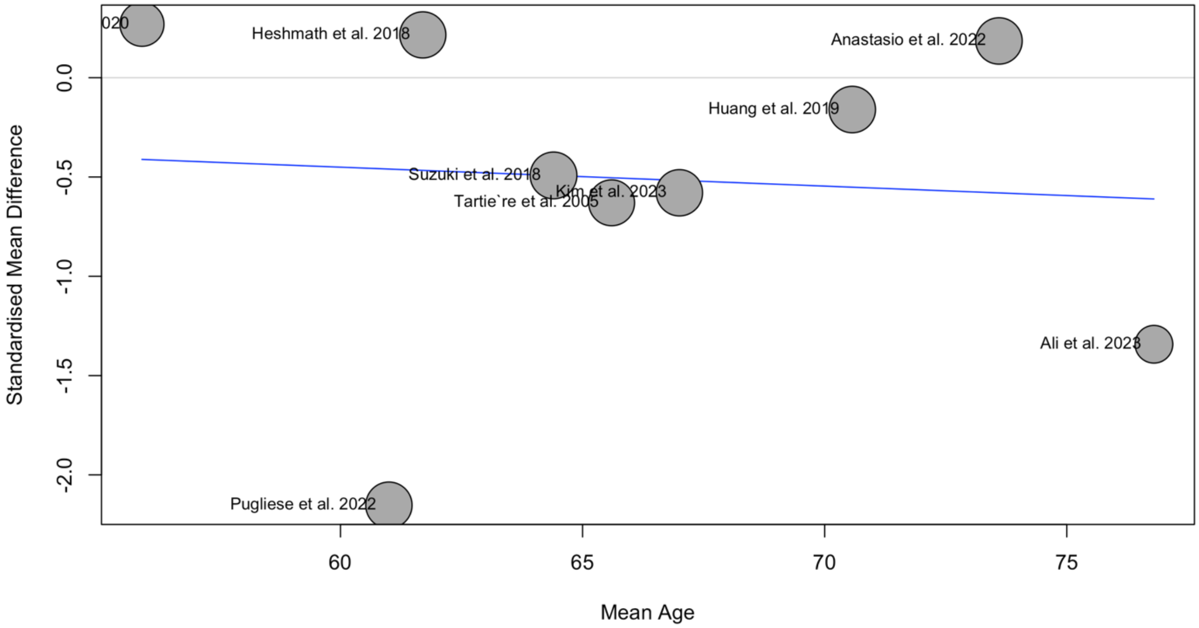

Fig S12:

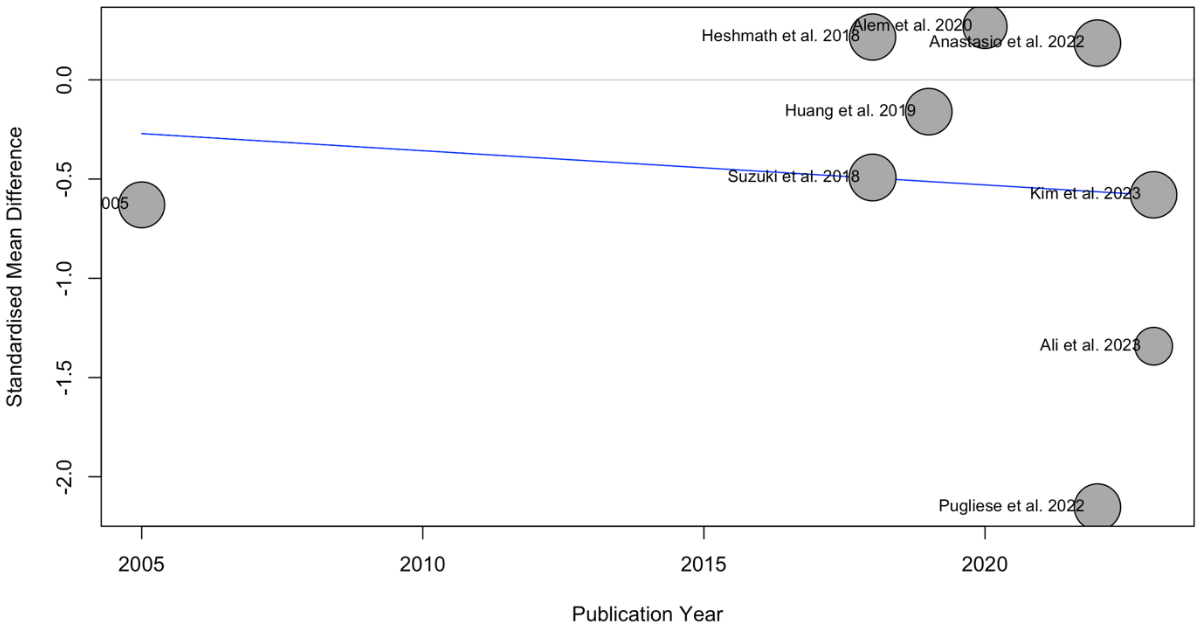

Fig S13:

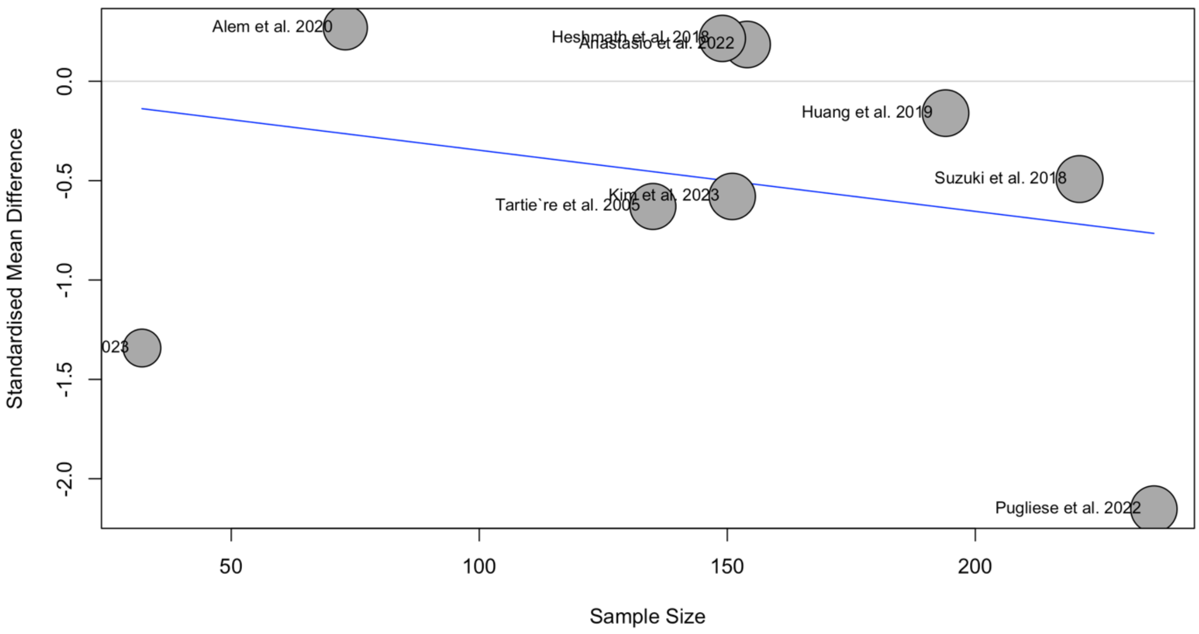

Fig S14:

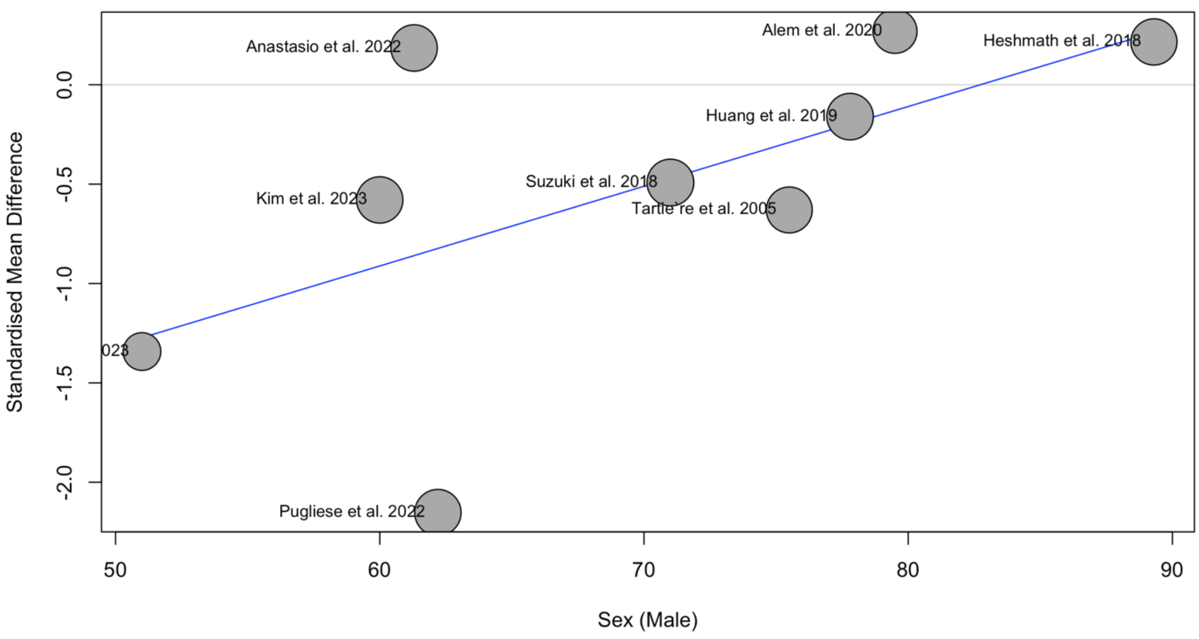

Supplementary Figure Legends:

Fig S1: Subgroup analysis of PWV in HF vs normal population based on the device of PWV measurement

Fig S2: Forest plot showing the PWV difference in HF vs normal population (removing outliers)

Fig S3: Sensitivity analysis of PWV in HF vs normal population

Fig S4: Bubble plot demonstrating the association between mean age and the PWV difference in HF vs normal population

Fig S5: Bubble plot demonstrating the association between year of publication and the PWV difference in HF vs normal population

Fig S6: Bubble plot demonstrating the association between sample size and the PWV difference in HF vs normal population

Fig S7: Bubble plot demonstrating the association between gender and the PWV difference in HF vs normal population

Fig S8: Forest plot showing the PWV difference in HFrEF vs HFpEF patients (removing outliers)

Fig S9: Subgroup analysis of PWV in HFrEF vs HFpEF patients based on the device of PWV measurement

Fig S10: Sensitivity analysis of PWV in HFrEF vs HFpEF patients

Fig S11: Bubble plot demonstrating the association between mean age and the PWV difference in HFrEF vs HFpEF patients

Fig S12: Bubble plot demonstrating the association between year of publication and the PWV difference in HFrEF vs HFpEF patients

Fig S13: Bubble plot demonstrating the association between sample size and the PWV difference in HFrEF vs HFpEF patients

Fig S14: Bubble plot demonstrating the association between gender and the PWV difference in HFrEF vs HFpEF patients
